# Central nervous system immune interactome is a function of cancer lineage, tumor microenvironment, and STAT3 expression

**DOI:** 10.1172/jci.insight.157612

**Published:** 2022-05-09

**Authors:** Hinda Najem, Martina Ott, Cynthia Kassab, Arvind Rao, Ganesh Rao, Anantha Marisetty, Adam M. Sonabend, Craig Horbinski, Roel Verhaak, Anand Shankar, Santhoshi N. Krishnan, Frederick S. Varn, Víctor A. Arrieta, Pravesh Gupta, Sherise D. Ferguson, Jason T. Huse, Gregory N. Fuller, James P. Long, Daniel E. Winkowski, Ben A. Freiberg, Charles David James, Leonidas C. Platanias, Maciej S. Lesniak, Jared K. Burks, Amy B. Heimberger

**Affiliations:** 1 Department of Neurological Surgery and; 2 Malnati Brain Tumor Institute of the Lurie Comprehensive Cancer Center, Feinberg School of Medicine, Northwestern University, Chicago, Illinois, USA.; 3 Miltenyi Biotec, Bergisch Gladbach, Germany.; 4 Department of General Surgery, University of Texas Medical Branch at Galveston, Galveston, Texas, USA.; 5 Department of Computational Medicine and Bioinformatics, University of Michigan, Ann Arbor, Michigan, USA.; 6 Department of Neurosurgery, Baylor College of Medicine, Houston, Texas, USA.; 7 The Jackson Laboratory, Farmington, Connecticut, USA.; 8 Department of Electrical and Computer Engineering, Rice University, Houston, Texas, USA.; 9 Translational Molecular Pathology Department,; 10 Neurosurgery Department,; 11 Neuropathology, and; 12 Biostatistics Department, The University of Texas MD Anderson Cancer Center, Houston Texas, USA.; 13 Visiopharm, Horsholm, Denmark.; 14 Robert H. Lurie Comprehensive Cancer Center and Division of Hematology-Oncology, Department of Medicine, Feinberg School of Medicine, Northwestern University, Chicago, Illinois, USA.; 15 Department of Medicine, Jesse Brown Veterans Affairs Medical Center, Chicago, Illinois, USA.; 16 Leukemia Department, The University of Texas MD Anderson Cancer Center, Houston Texas, USA.

**Keywords:** Immunology, Oncology, Brain cancer, Innate immunity, T cells

## Abstract

**BACKGROUND:**

Immune cell profiling of primary and metastatic CNS tumors has been focused on the tumor, not the tumor microenvironment (TME), or has been analyzed via biopsies.

**METHODS:**

En bloc resections of gliomas (*n* = 10) and lung metastases (*n* = 10) were analyzed via tissue segmentation and high-dimension Opal 7-color multiplex imaging. Single-cell RNA analyses were used to infer immune cell functionality.

**RESULTS:**

Within gliomas, T cells were localized in the infiltrating edge and perivascular space of tumors, while residing mostly in the stroma of metastatic tumors. CD163^+^ macrophages were evident throughout the TME of metastatic tumors, whereas in gliomas, CD68^+^, CD11c^+^CD68^+^, and CD11c^+^CD68^+^CD163^+^ cell subtypes were commonly observed. In lung metastases, T cells interacted with CD163^+^ macrophages as dyads and clusters at the brain-tumor interface and within the tumor itself and as clusters within the necrotic core. In contrast, gliomas typically lacked dyad and cluster interactions, except for T cell CD68^+^ cell dyads within the tumor. Analysis of transcriptomic data in glioblastomas revealed that innate immune cells expressed both proinflammatory and immunosuppressive gene signatures.

**CONCLUSION:**

Our results show that immunosuppressive macrophages are abundant within the TME and that the immune cell interactome between cancer lineages is distinct. Further, these data provide information for evaluating the role of different immune cell populations in brain tumor growth and therapeutic responses.

**FUNDING:**

This study was supported by the NIH (NS120547), a Developmental research project award (P50CA221747), ReMission Alliance, institutional funding from Northwestern University and the Lurie Comprehensive Cancer Center, and gifts from the Mosky family and Perry McKay. Performed in the Flow Cytometry & Cellular Imaging Core Facility at MD Anderson Cancer Center, this study received support in part from the NIH (CA016672) and the National Cancer Institute (NCI) Research Specialist award 1 (R50 CA243707). Additional support was provided by CCSG Bioinformatics Shared Resource 5 (P30 CA046592), a gift from Agilent Technologies, a Research Scholar Grant from the American Cancer Society (RSG-16-005-01), a Precision Health Investigator Award from University of Michigan (U-M) Precision Health, the NCI (R37-CA214955), startup institutional research funds from U-M, and a Biomedical Informatics & Data Science Training Grant (T32GM141746).

## Graphical abstract

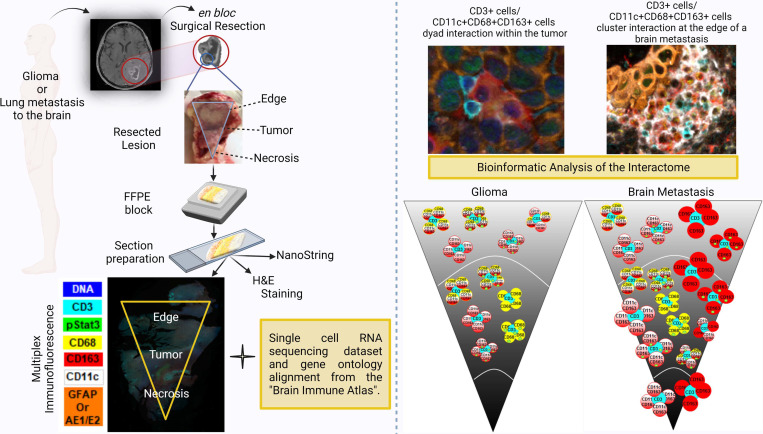

## Introduction

Immune response to the presence of brain tumors results from the lymphatic drainage of tumor antigens to the cervical lymph nodes (1), where professional antigen-presenting cells (APCs), such as DCs, present them to T cells (2). Subsequent to this interaction and presumed activation, the T cells traffic to the tumor. However, immune phenotyping has revealed that intratumoral T cells lack antitumor function and are exhausted (3), possibly as a result of chronic T cell stimulation with weak tumor antigens (4). The process by which lymph node–activated T cells become inactive or exhausted once within tumor is not completely understood. It has been suggested that T cell activation within the tumor microenvironment (TME), rather than distally in lymph nodes, may reduce the extent of T cell exhaustion and enable tumor cytotoxicity (5). Alternatively, an APC event within the TME may be needed for full T cell effector functions (6).

Immune response to the presence of glioma is known to be suppressed by tumor cell–secreted factors that activate the signal transducer and activator of transcription 3 (STAT3) pathway (7). Phosphorylation of STAT3 (p-STAT3) in macrophages inhibits their activation (8), including their role in promoting an inflammatory response (9). p-STAT3 in DCs decreases their expression of MHC II, CD80, CD86, and IL-12, which limits DC stimulation of T cells and T cell antitumor activity (10). p-STAT3 also blocks CX3CR1^+^CD11c^+^ DC stimulation of T cell proliferation by reducing the expression of CD80/CD86 (11).

The protumor effects resulting from STAT3 activation in immune cells can be mitigated through the use of p-STAT3 inhibitors (12). Recently, we showed that DC–T cell cluster events are promoted in the glioma TME by combined radiation and p-STAT3 inhibitor (WP1066) treatments that confer long-term survival to animals with intracranial tumors (13). These effects are due, in part, to the inhibition of p-STAT3 activities that result in the inhibition of FoxP3 expression in Tregs (14), increasing IFN-γ production and accumulation within tumors (15), inhibiting PD-L1 expression (16), and blocking M2 skewing (17) that inhibits T cell proliferation. STAT3 phosphorylation and activation in reactive astrocytes has also been associated with tumor metastasis to brain (18), possibly by contributing to a microenvironment that attracts tumor cells from distal locations (19–22).

In this study, we used 7-color multiplex staining, high-resolution spectral imaging microscopy, and geospatial algorithm analysis to examine immune cell distribution and interactions in different regions of individual tumors, including regions of necrosis and tumor as well as at the brain-tumor interface or infiltrating edge. Compared with the recent studies that explore the TME and its immune composition through multiple biopsies taken from different disconnected locations (23–26), our approach preserves tissue sample structure, orientation, and architecture throughout all areas of the TME, from the edge to the tumor and to the necrotic core, thereby enabling the analysis of cellular interactions in continuum within the TME.

## Results

### Cohort.

Primary tumors, most of which were GBM, and brain metastases from lung cancer were included in this study (Table 1).

### Transcriptional analysis reveals differences in immune cell infiltration based on location and cancer type.

NanoString and segmentation analysis for 770 immune genes were used to determine the types of immune cells present within different tumor regions. The top upregulated immune genes in GBM necrotic cores were associated with macrophages and included the CD163 marker; chemotactic factors (such as *CCL18* and *SAA1*); and the phagocytosis stimulatory factors (such as IL-8 and *MARCO*) (Supplemental Figure 1; supplemental material available online with this article; https://doi.org/10.1172/jci.insight.157612DS1). In comparison to data associated with the analysis of other tumor regions, necrotic regions showed decreased RNA levels for the expression of GBM antigens (such as *IL13RA2* and *MAGEB2*), DC markers (such as *LILRA4*), as well as the expression of immune stimulatory processes, including MHC, IFN, IL-12, TNF, and ICOS (Supplemental Figure 1). The infiltrating edge of GBM, relative to tumor in total, was enriched with RNAs for stimulators of NK cytotoxicity (i.e., CD244, the fractalkine receptor for immune cells); chemokines for thymocytes and DCs; and immune stimulatory IL-12 receptors. Regardless of primary versus metastatic tumor subtype, monocytes and monocyte-derived populations, such as macrophages, were the most abundant immune cell type designated by RNA analysis. In necrotic cores, especially those in GBM, macrophages were polarized to the M0 and M2 phenotypes (Supplemental Figure 1), consistent with results from our previous studies (27, 28). Brain metastases from lung cancers showed significant M1 phenotype macrophages at brain-tumor interface, as well as in intratumoral regions, and tumor necrosis. Notably, DCs and activated T cell levels were of low frequency in GBM relative to lung metastasis, while the latter was highly infiltrated with CD3^+^ T cells at the brain-tumor interface. In summary, we noted distinct inter- and intratumoral immune gene signatures, with macrophages being the major immune cell population in the TME, especially in the necrotic core, regardless of cancer lineage.

### Multiplex immunofluorescence observations along the TME continuum.

**Figure 1:**
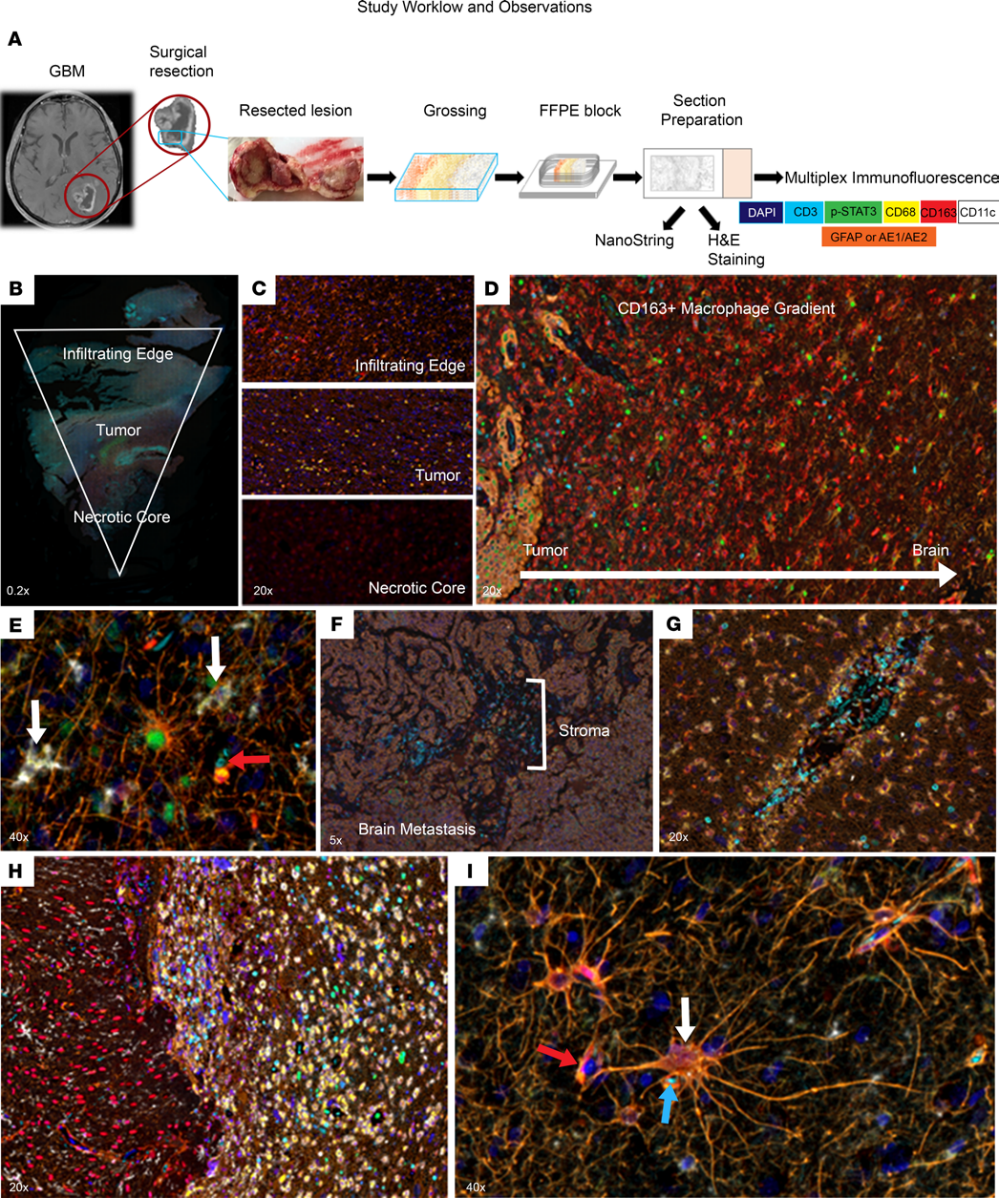
Study workflow and key observations of the TME. (**A**) Study workflow. After en bloc gross total resection of diagnosed brain tumor, the surgeon cuts a wedge that includes an infiltrating edge or normal brain (in the case of brain metastasis), tumor, and necrotic core. Formalin-fixed paraffin-embedded slides were prepared subsequently for analysis. The 7-color Opal multiplex-staining panel includes DAPI (dark blue nuclei), CD3 (Opal 480, cyan blue), p-STAT3 (Opal 520 [Akoya Biosciences], green), CD68 (Opal 570 [Akoya Biosciences], yellow), CD163 (Opal 690 [Akoya Biosciences], red), CD11c (Opal 780 [Akoya Biosciences], white), and GFAP or AE1/AE2 (Opal 620 [Akoya Biosciences], orange). (**B**) Representative multiplex imaging of a whole-mount section of a glioblastoma (GBM), spanning from infiltrating edge to necrotic core, visualized using Phenochart software (original magnification, ×0.2). (**C**) Representative images of 3 different regions of glioma TME (infiltrating edge, tumor, and necrotic core) (original magnification, ×20). There is a predominance of CD68^+^ cells within the tumor area in contrast to the edge and necrotic core where CD163^+^ macrophages predominate. (**D**) Gradient of CD163^+^ macrophages in which the density is highest near brain metastasis and decreases toward normal brain (original magnification, ×20). (**E**) p-STAT3^+^ reactive astrocytes at the infiltrating edge of glioma. The 2 white arrows point to CD11c^+^CD68^+^ microglia. The red arrow denotes red blood cells (RBCs) in which the signal from Opal 480 (cyan) was differentiated from the T cells by the absence of DAPI (original magnification, ×40). (**F**) CD3^+^ T cell infiltration of brain metastasis observed in the stroma of the tumor (original magnification, ×5). (**G**) CD3^+^ T cells, within the tumor, have extravasated from the vessel but are located adjacent to the vessel, and CD163^+^ macrophages as well as CD68^+^ monocytes line the vasculature wall in GBM. Some RBCs are also present inside the vessel (original magnification, ×20). (**H**) CD3^+^ T cell infiltration shown at the gliotic plane (infiltrating edge) in low-grade glioma (original magnification, ×20). (**I**) p-STAT3^–^ nonreactive astrocyte (white arrow) in close proximity to a CD3^+^p-STAT3^–^ T cell (blue arrow) and a CD163^+^p-STAT3^–^ macrophage (red arrow) located in the normal brain/infiltrating edge in low-grade glioma (original magnification, ×40).

Glioma (*n* = 10) and CNS lung metastasis (*n* = 10) en bloc resections were oriented on slides as whole-mount wedges spanning 3 areas, including the brain-tumor interface/infiltrating edge, tumor, and necrotic core. Specimens were interrogated using a 7-color multiplex immunofluorescence panel that included lineage markers CD3 (T cells), CD68 (monocyte-derived cells), CD11c (APCs); the CD163 macrophage phenotype marker; immunosuppressive p-STAT3; and tumor cells markers, GFAP (glioma) and AE1/E2 (lung brain metastasis). Specimens were also stained for nuclear DAPI (Figure 1, A–C). Analysis of results from tumor-infiltrating edge to necrotic core revealed substantial regional differences in immune cell composition (Figure 1, B and C). In normal brain parenchyma, occasional CD3 T cells and scavenger receptor CD163^+^ macrophages were identifiable, but other immune cell populations identified with the multiplex panel were virtually absent. CD163^+^ macrophages were frequent at the infiltrating edge and necrotic core of tumors (Figure 1C). Their highest concentration was consistently at the brain-tumor interface/infiltrating edge of primary and metastatic tumors, but with diminishing levels in transition further outward into nonneoplastic brain (Figure 1D). Reactive astrocytes, as reflected by p-STAT3 nuclear expression (29, 30), could be identified at the infiltrating edge of tumors (Figure 1E). In this same location, isolated CD11c^+^CD68^+^ amoeboid cells that may be reactive microglia were also evident (31–33). T cells were much less frequent across tumors relative to myeloid-derived lineages, irrespective of primary versus metastatic tumor type. The highest concentration of T cells was found in the stroma of brain metastases (Figure 1F). In GBM, T cells were mostly found in perivascular regions and showed strong nuclear p-STAT3 expression (Figure 1G), suggesting their lack of cytotoxic effector functions and/or identity as Tregs. T cells could also be found at the infiltrating edge in gliomas that surgeons refer to as the gliotic plane (Figure 1H). Astrocytes could be found associating with CD163^+^ macrophages and CD3^+^ T cells in the normal brain (Figure 1I); these are likely podocytes, that are part of the blood-brain barrier capillary system. p-STAT3^+^ reactive astrocytes do not usually associate with CD163^+^ macrophages and the CD3^+^ T cells (Figure 1E).

### Immune cell composition and distribution between cancer lineages.

**Figure 2:**
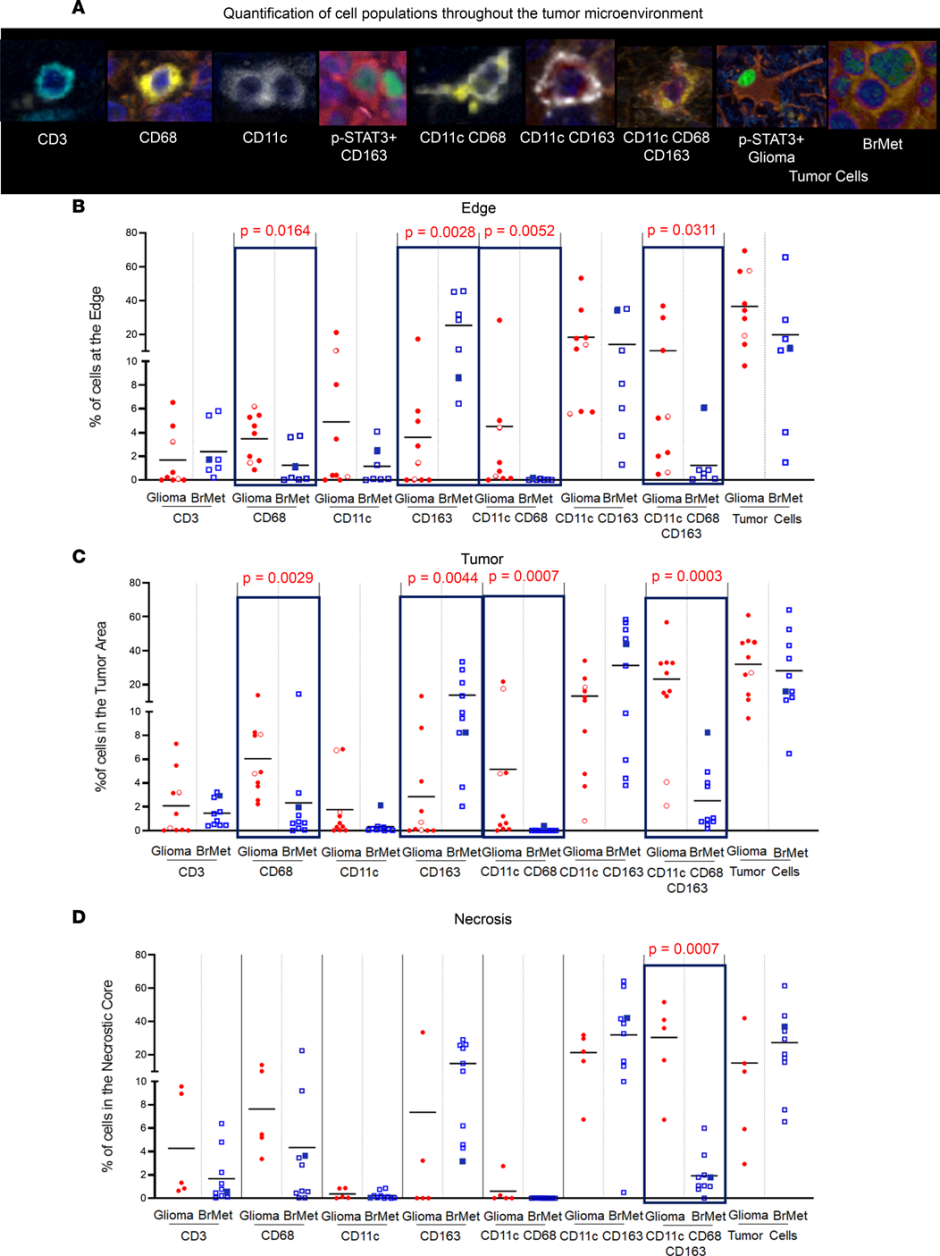
Representative images of each cell type and dot plots representing the percentages of the different cell populations in the 3 different regions. (**A**) Representative images of the different cell populations: CD3^+^p-STAT3^–^ T cell, CD68^+^p-STAT3^–^ monocyte-derived cells, CD11c^+^p-STAT3^–^ DCs, CD163^+^p-STAT3^+^ macrophages, CD11c^+^CD68^+^p-STAT3^–^ microglia, CD11c^+^CD163^+^p-STAT3^–^ DCs, CD11c^+^CD68^+^CD163^+^p-STAT3^–^ APCs, and tumor cells heterogeneously expressing nuclear p-STAT3. Original magnification, ×20 (CD3, CD68, p-STAT3^+^CD163, and CD11cCD68CD163); ×30 (CD11c, CD11cCD68, CD11cCD163, p-STAT3^+^ Glioma, and BrMet). (**B**) Edge, (**C**) tumor, and (**D**) necrosis in gliomas versus brain metastases (BrMet). Each solid red circle represents a GBM specimen, and the red empty circles represent astrocytoma grade II. The empty blue squares represent adenocarcinoma lung cancer brain metastasis, and the solid blue squares represent squamous lung cancer metastasis. Two-sample Wilcoxon’s rank-sum (unpaired) exact test to compare between glioma and metastasis, and 1-way repeated-measures ANOVA to compare the percentages for each marker across regions, were performed. Statistically significant comparisons (*P* ≤ 0.05) are highlighted with black rectangles.

Differences in the frequency of immune cell populations (Figure 2A) were analyzed based on tumor type, location within the TME (brain-tumor interface/infiltrating edge, tumor, and necrosis), and p-STAT3 expression. In the glioma cohort, 5 of 10 specimens had distinguishable areas of necrosis, and 9 of 10 had clearly discernible infiltrating edges. In the brain metastasis, 7 of 10 had distinguishable brain adjacent to tumor (i.e., brain-tumor interface), and all the specimens (10 of 10) had an identifiable area of necrosis (Figure 2).

Overall, T cell frequencies were similar between gliomas and brain metastases, regardless of location within the TME (Figure 2, B–D). In contrast, CD68^+^ monocyte-derived cells and CD11c^+^CD68^+^ cells were more frequent at the infiltrating edge (*P* = 0.0164 and *P* = 0.0052, respectively) as well as within the tumor area (*P* = 0.0029 and *P* = 0.0007, respectively) of glioma specimens relative to brain metastases (Figure 2, B and C). The finding of enrichment of CD11c^+^CD68^+^ cells (potentially microglia) (31) at the glioma edge is consistent results from a prior study (25). CD163^+^ macrophages were more abundant within the tumor area and at the edge of brain metastases (*P* = 0.0044 and 0.0028, respectively). CD11c^+^CD68^+^CD163^+^ APCs were the immune population found to be preferentially enriched in gliomas relative to metastases, irrespective of the tumor compartment being considered (edge, *P* = 0.0311; tumor, *P* = 0.0003; necrosis, *P* = 0.0007) (Figure 2, B–D). This immune cell may be a DC3 capable of producing high levels of IL-12 and stimulating type 1 T cell polarization (34).

**Figure 3:**
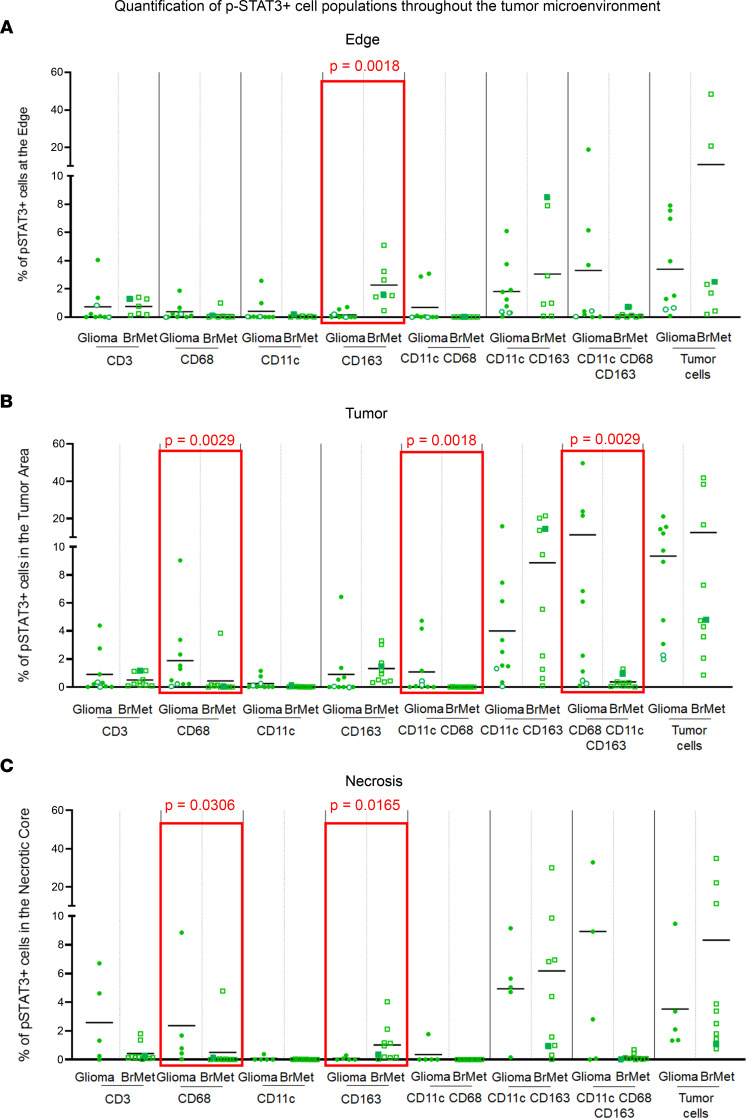
Dot plots representing the percentages of p-STAT3^+^ cell populations in the 3 different areas. (**A**) Edge, (**B**) tumor, and (**C**) necrosis in gliomas versus brain metastases (BrMet). Each solid green circle represents a GBM, and the green empty circles represent astrocytoma grade II. The green empty squares represent adenocarcinoma lung cancer brain metastasis specimens, and the solid green squares represent squamous lung cancer metastasis. Two-sample Wilcoxon’s rank-sum (unpaired) exact test to compare between glioma and metastasis, and 1-way repeated-measures ANOVA to compare the percentages for each marker across regions, were performed. Statistically significant comparisons (*P* ≤ 0.05) are represented within red rectangles.

When the immune cell phenotypes were analyzed with respect to nuclear p-STAT3 expression, preferential association with CD163^+^ cells at the edge of metastases were evident (*P* = 0.0018) (Figure 3A). Intratumoral CD68^+^ (*P* = 0.0029), CD11c^+^CD68^+^ (*P* = 0.0018) and CD11c^+^CD68^+^CD163^+^ (*P* = 0.0029) cells were more likely to express p-STAT3 in gliomas (Figure 3B). CD68^+^p-STAT3^+^ (*P* = 0.0306) and CD163^+^p-STAT3^+^ (*P* = 0.0165) cells were significantly enriched in regions of necrosis in gliomas and brain metastases, respectively (Figure 3C).

### The immune interactome as a function of tumor type and TME.

**Figure 4:**
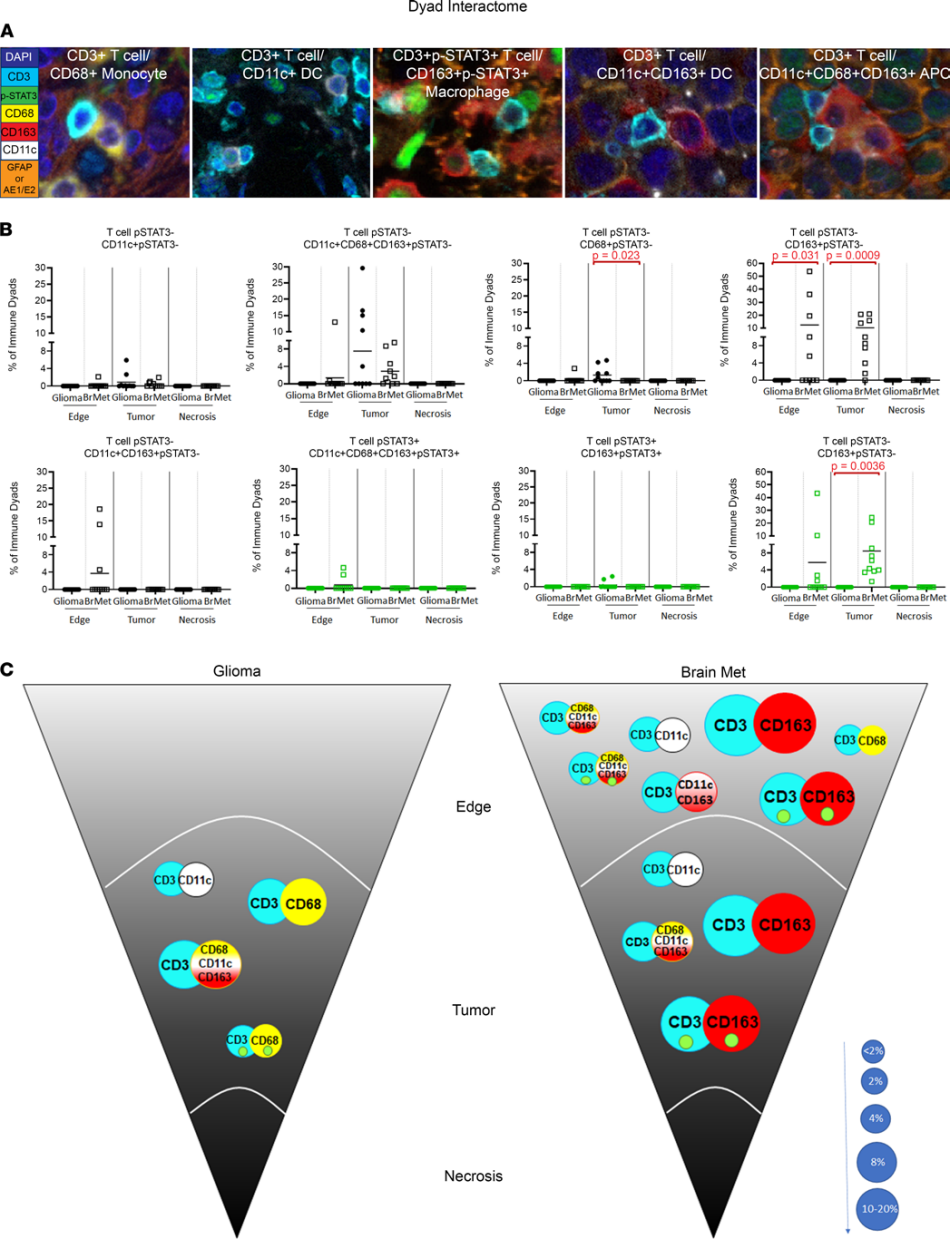
T cell dyad interactions within the TME. (**A**) Examples of the various dyad interactions occurring in the TME of gliomas and metastasis (original magnification, ×40). From left to right, a CD3^+^p-STAT3^–^ T cell interacting with a CD68^+^p-STAT3^–^ monocyte-derived cell, a CD3^+^p-STAT3^–^ T cell with CD11c^+^p-STAT3^–^ DC, a CD3^+^p-STAT3^+^ T cell with a CD163^+^p-STAT3^+^ macrophage, a CD3^+^p-STAT3^–^ T cell with CD11c^+^CD163^+^p-STAT3^–^ APCs, and a CD3^+^p-STAT3^–^ T cell with CD11c^+^CD68^+^CD163^+^p-STAT3- APCs. (**B**) Scattered dot plots showing the probability (%) of each of the dyad interactions identified throughout the 3 regions of the TME (edge, tumor, and necrosis) in gliomas versus brain metastasis (BrMet) based on G-function analysis. Each solid circle represents a glioma, and the empty squares represent lung cancer brain metastasis specimens. The color black represents p-STAT3^–^ dyads, and the color green represents p-STAT3^+^ dyads. Two-sample Wilcoxon’s rank-sum (unpaired) exact test to compare between glioma and metastasis, and 1-way repeated-measures ANOVA to compare the probability for each dyad interaction across regions, were performed. Statistically significant comparisons (*P* ≤ 0.05) are highlighted in red. (**C**) Schema summarizing the location-dependent distribution of the immune dyad interactions in gliomas versus brain metastasis (met) in the 3 regions of the TME. Inset green circles designate positive nuclear expression of p-STAT3. The size of each circle reflects the mean probability displayed in **B** within the TME. The size of the circles correlates with the frequency of the interaction, represented by the size scale on the bottom right.

Analysis of specimen multiplex immunofluorescence staining revealed interactions of T cells with other immune cells, such as CD68^+^ monocyte-derived cells, CD11c^+^ APCs, CD163^+^ macrophages, CD11c^+^CD68^+^ possible microglia, and CD11c^+^CD68^+^CD163^+^ APCs. We used 15 μm (35) as the upper limit of distance indicating dyad interaction between cells (Figure 4A). First, T cell interactions with CD68^+^, CD163^+^, CD11c^+^CD163^+^, and CD11c^+^CD68^+^CD163^+^ cells were not observed in areas of necrosis for either tumor type. Second, despite similar T cell frequencies in gliomas and brain metastases (Figure 2, B–D), glioma T cells preferentially interacted with CD68^+^p-STAT3^–^ monocyte-derived cells within tumor relative to that observed for brain metastases (*P* = 0.023) (Figure 4B). In contrast, T cells in brain metastases were much more likely to associate with CD163^+^pSTAT3^–^ macrophages within tumor (*P* = 0.009) and at the edge (*P* = 0.031), and with CD163^+^pSTAT3^+^ macrophages within tumor (*P* = 0.036) (Figure 4B). Additionally, in brain metastases, T cells were observed interacting with other types of immune cells, such as CD68^+^ monocyte-derived cells, CD11c^+^ APCs, and CD11c^+^CD68^+^CD163^+^ APCs (Figure 4C). p-STAT3 expression was consistently observed among immune dyad interactions, regardless of TME location or tumor type.

### Immune cluster interactions predominate in brain metastases.

**Figure 5:**
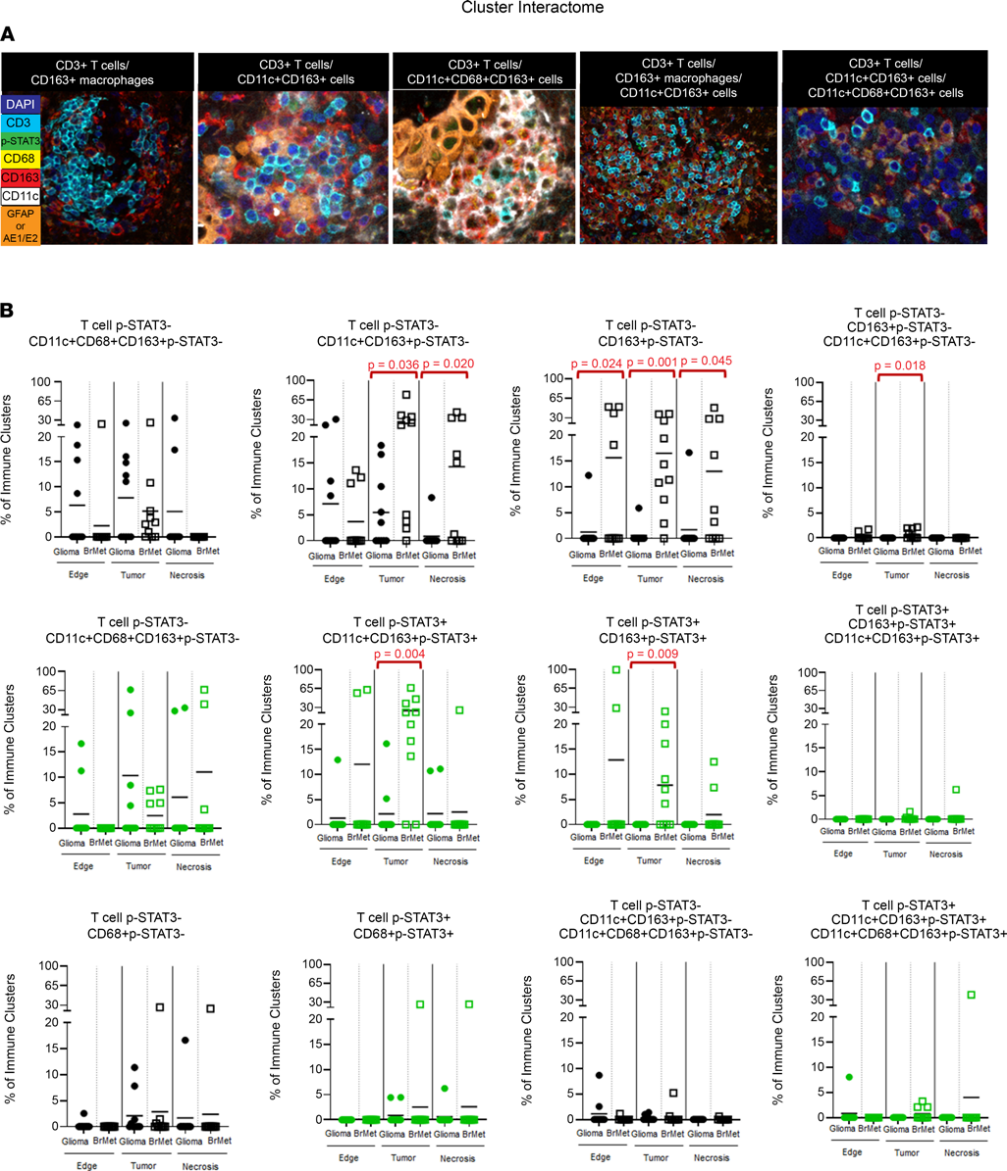
T cell cluster interactions within the TME. (**A**) Examples of the various cluster interactions occurring in the TME of gliomas and metastasis (original magnification, ×20). From left to right, CD3^+^ T cells with CD163^+^ macrophages in the stroma of the tumor in brain metastasis, CD3^+^ T cells with CD11c^+^CD163^+^ cells, CD3^+^ T cells with CD11c^+^CD68^+^CD163^+^ cells at the edge of brain metastasis, and CD3^+^ T cells with CD163^+^ macrophages and CD11c^+^CD163^+^ cells. (**B**) Scattered dot plots showing the frequency (%) of the different cluster interactions identified with the 3 regions of the TME (edge, tumor, and necrosis) in gliomas versus brain metastasis (BrMet) obtained through cross pair count analysis. Each solid circle represents a glioma, and the empty squares represent lung cancer brain metastasis specimens. The color black represents p-STAT3^–^ clusters, and the color green represents p-STAT3^+^ clusters. Two-sample Wilcoxon’s rank-sum (unpaired) exact test to compare between glioma and metastasis, and 1-way repeated-measures ANOVA to compare the percentages for cluster interaction across regions, were performed. Statistically significant comparisons (*P* ≤ 0.05) are highlighted in red.

**Figure 6:**
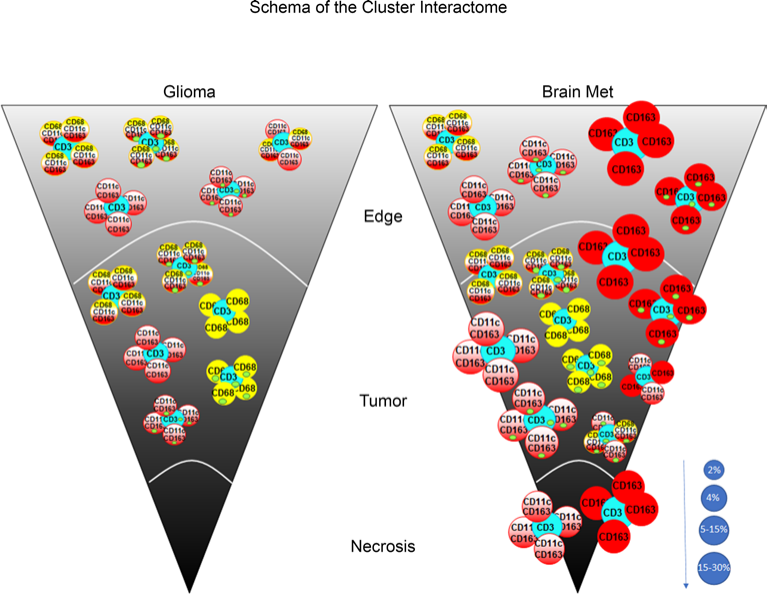
Location-dependent distribution of the immune cluster interactions in gliomas versus lung metastasis in the 3 regions of the TME (edge, tumor, and necrosis). Inset green circles designate positive nuclear expression of p-STAT3. The size of each circle reflects the mean frequency displayed in Figure 5B within the TME. The size of the circles correlates with the frequency of the interaction represented by the size scale on the bottom right.

Cell cluster interactions within the TME were also identified, usually at the infiltrating edge of tumors or within the stroma of brain metastases (Figure 5A). Throughout the TME (adjacent brain/infiltrating edge, tumor, and regions of necrosis), CD3^+^p-STAT3^–^ T cell/CD163^+^pSTAT3^–^ macrophage clusters were significantly more common in brain metastases (*P* = 0.024, *P* = 0.01, and *P* = 0.045, respectively) (Figure 5B and Figure 6). CD3^+^p-STAT3^–^ T cells clustering with CD11c^+^CD163^+^p-STAT3^–^ cells in tumoral and necrotic regions of metastases (*P* = 0.036 and *P* = 0.020, respectively) were also evident (Figure 5B). CD163^+^p-STAT3^+^ clustering also occurred and was significantly more frequent within tumor areas of metastases than in gliomas (Figure 5B). An additional type of cluster interaction that was significantly higher within the tumor area of brain metastases involves CD3^+^p-STAT3^–^ T cells with both CD163^+^p-STAT3^–^ macrophages and CD11c^+^CD163^+^p-STAT3^–^ cells (*P* = 0.018) (Figure 5B). Multiple other types of clusters are present in gliomas and metastases but with no significant difference between cancer types. Such interactions include CD3^+^ T cells with CD11c^+^CD68^+^CD163^+^ cells, CD3^+^ T cells with CD68^+^ monocyte-derived cells, and CD3^+^ T cells with CD11c^+^CD163^+^ and CD11c^+^CD68^+^CD163^+^ cells (Figure 5B and Figure 6).

### Transcriptomics and gene ontology alignment.

**Figure 7:**
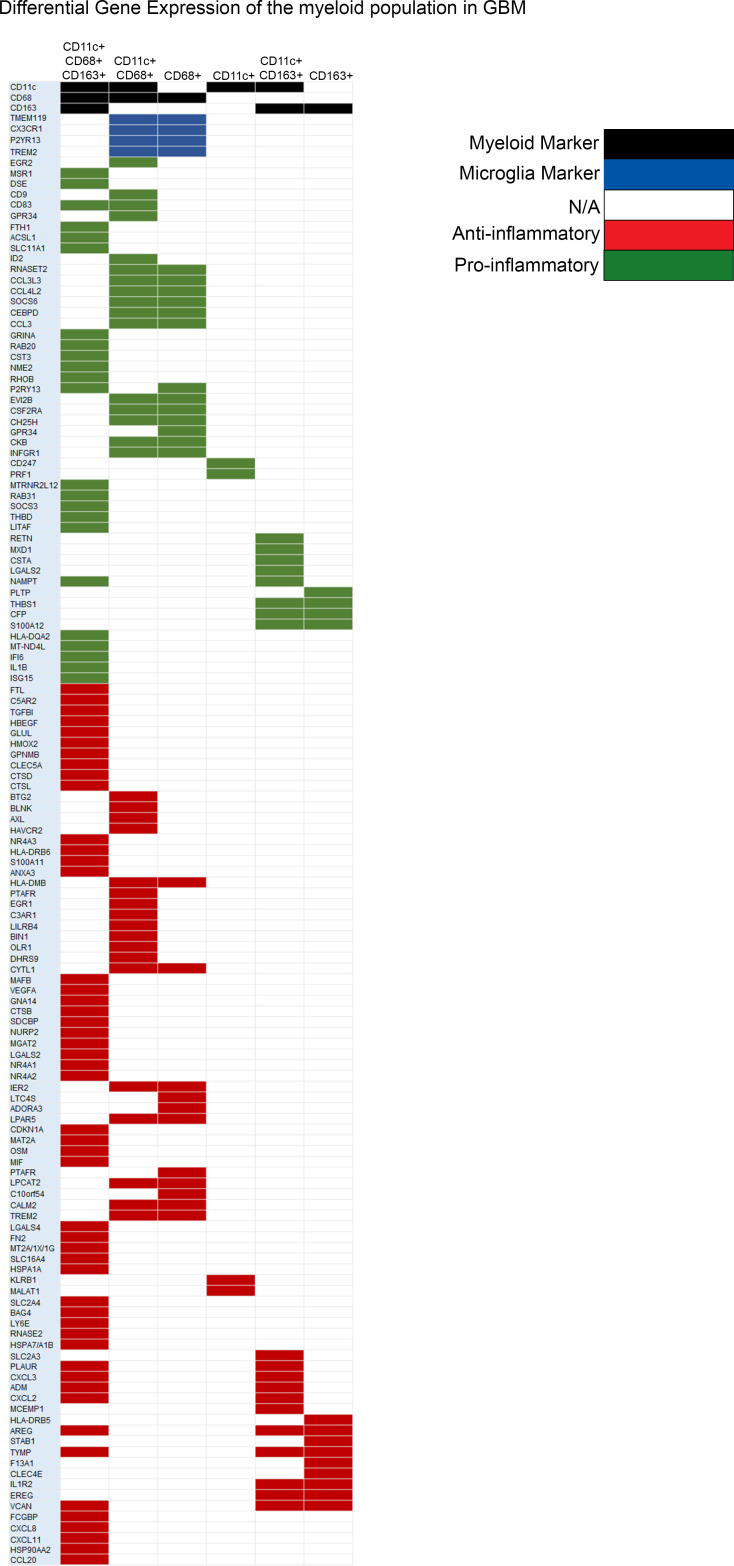
Heatmap of the differential gene expression of the various myeloid populations based on scRNA-seq analysis of the CD45^+^ immune population in GBM. *n* = 7. Red denotes immunosuppressive genes, and green denotes proinflammatory genes. White indicates NA, black indicates markers of myeloid cells, and blue indicates microglia markers. The RNA-Seq data set was processed using the associated script_scRNA.seq.R processing script, and differential expression was performed using the FindMarkers tool in Seurat (version 3.2.3). All differentially expressed genes are statistically significant with adjusted *P* < 0.05 and average log fold change of more than 0.

**Figure 8:**
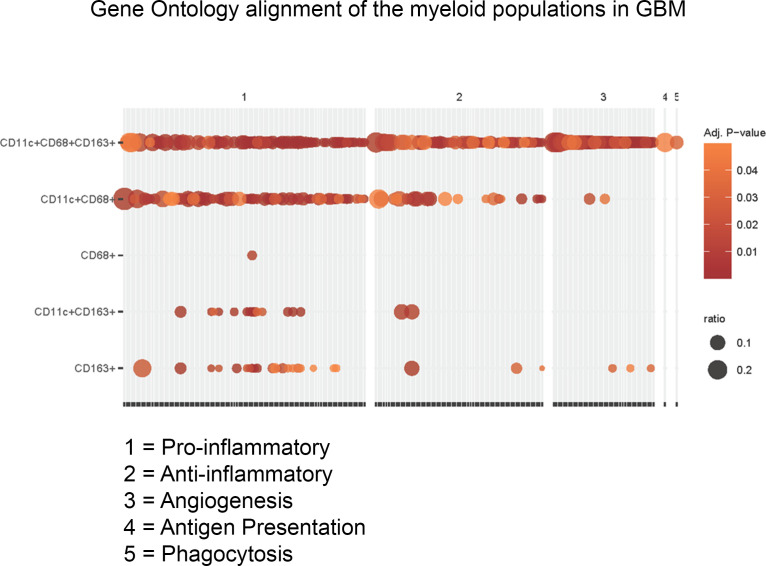
GO enrichment heatmap of the myeloid populations based on the transcriptomic data of the CD45^+^ immune population in GBM, performed using the topGO package’s “classic” algorithm (version 2.42.0). *n* = 7. *P* values from this analysis were adjusted using the Benjamini-Hochberg procedure (with adjusted *P* < 0.05 representing the significantly enriched GO terms). The number 1 represents proinflammatory functions, 2 represents antiinflammatory functions; 3 represents angiogenesis; 4 represents antigen presentation; and 5 represents phagocytosis.

To define the potential functionality of the different immune populations identified, a bioinformatic single-cell RNA-Seq (scRNA-Seq) analysis was performed on the CD45^+^ (a general marker for hematopoietic cells) immune populations from patients with GBM (*n* = 7) (36). The functional aspect of each gene is listed in the Supplemental Table 1. This analysis revealed elevated immunosuppressive gene expression in CD163^+^ macrophages that promote angiogenesis (37, 38), inhibit IL-1 signaling (39), and exert wound healing activity (40–42) (Figure 7). The remaining immune cells, such CD68^+^ monocyte-derived cells, CD11c^+^ APCs, CD11c^+^CD68^+^ cells (potential microglia), and CD11c^+^CD163^+^ APCs, expressed various proinflammatory and antiinflammatory genes (Figure 7), implying complex immunological heterogeneity (Figure 8). Both CD68^+^ and CD11c^+^CD68^+^ cells expressed markers such as *CX3CR1* (43), *TMEM119* (44, 45), *P2RY13* (46, 47), and *TREM2* (48, 49), which typically define microglia. Notably, CD11c^+^ APCs expressed the CD247 coding gene that is a T cell surface receptor responsible for T cell receptor activation and signaling (50–52), indicating potential cytotoxic effector function (Figure 7). CD11c^+^CD68^+^CD163^+^ cells significantly expressed proinflammatory genes related to cell killing; phagocytosis (*MSR1*, etc.); IL-1β, IFN-γ (*NAMPT*), and TNF-α (*LITAF*) signaling; and antigen presentation. However, these cells also significantly expressed antiinflammatory genes presumed to define M2 polarized macrophages such as TGF-β (53–55) (Figures 7 and 8).

## Discussion

In this immune topographical atlas study, we used multiplex immunofluorescence staining and RNA-Seq to characterize immune cell compositions (by determining the abundance of different immune cell subpopulations), distributions, and interactions, throughout the TME in linearity, in primary and metastatic brain tumors. To date, nearly all brain tumor immune profiling studies have relied on the deconvolution of data obtained from bulk tumor samples (25, 26), which does not provide information on cell subtype variability and interactions across the tumor landscape. En bloc surgical resection technique and subsequent tissue processing has also enabled a comparison of immune cell composition among regions of tumor, necrosis, and the interface or edge of advancing tumor with normal brain.

Several findings have emerged from this analysis, including the determination (a) that T cells are typically identified in the perivascular space of the tumor and are not homogeneously distributed; (b) that a CD163 macrophage migration gradient is formed in the infiltrating adjacent brain of both gliomas and brain metastasis; (c) of the predominance of innate immune cells in areas of necrosis, likely clearing necrotic debris; (d) that T cells can be enriched at the tumor edge, and they may be missed during routine banking processes; (e) of marked differences in immune cell interactions when comparing primary versus metastatic tumors; and (f) of a disparity in immune cell dyads and clusters among cancer types, with both being more common in metastases. Of relevance to the latter of these observations, brain metastases (56–60), but not primary gliomas (61), respond to immune checkpoint inhibitors. In combination with our results, this suggests that the presence of dyads and clustering could serve as a biomarker, indicating immune checkpoint therapy responsiveness of individual tumors (24, 62), which is an area of future investigation, similar to the ongoing investigations in other cancers (63, 64).

Notably, brain metastasis specimens were highly infiltrated with CD163^+^ immunosuppressive macrophages. Multiple studies have shown a special role of these cells in promoting metastasis to the brain and their contribution to an immunosuppressive microenvironment that inhibits T cell activation (19, 22). As such, therapeutic targeting of this immune population might be key to promoting T cell activation and effective immune response against metastases.

Our study also focused on the role of p-STAT3 in the immunobiology of brain tumors. The immunofluorescence markers used were prioritized, in part, owing to the testing of a small-molecule inhibitor of p-STAT3 in phase I trials (NCT01904123; NCT04334863). Applying the immune cell markers used here to the analysis of tumors from patients enrolled in these trials would prove informative, regarding inhibitor effects on immune cell interactions and patient response to treatment. In a preclinical model of glioma, immune cluster interactions are not present within the TME at baseline but can be therapeutically induced with the combination of radiation therapy and the p-STAT3 inhibitor WP1066 (13). Using window-of-opportunity clinical trial design, the therapeutic induction of these immune clusters could be assessed with this multiplex panel in people with glioma treated with this combinatorial strategy.

In contrast to T cells, which have well-defined lineage markers, the myeloid cell population in brain tumors is much more heterogenous. This complicates the identification of distinct innate immune cell populations and determining their interactions. Fortunately, the acquisition and analysis of transcriptomic data from tumors analyzed by multiplex immunofluorescence has provided some clarification of immune cell types and functions. Specifically, scRNA-Seq analysis of the CD45^+^ immune population in GBM showed this population as expressing gene signatures reflective of both immune activation and immune suppression, that, in sum, demonstrate and provide evidence of the complex heterogeneity of the myeloid compartment that might be dependent upon the location within the TME. This needs additional thorough analysis for a better understanding.

One other limitation in this study is the limited number of cases per group that restricts our capabilities to demonstrate specific characteristics for cancer types and subtypes. This type of analysis does not lend itself to high-volume throughput of specimens (65). In fact, patients with high-grade cancer are exposed to multiple regimens of therapies, which makes it a challenge to have comparable patient groups with higher number of patients between primary and metastatic brain cancers. Furthermore, not all patients with brain metastasis are eligible to undergo surgical resection. Multiple solution strategies are ongoing to overcome these challenges for future studies.

The emergence of single-cell analysis and CyTOF data from different glioma subtypes is likely to change conventional thinking about immune cell relationships and function in brain tumors. Our study marks a starting point for this type of analysis, for which we envision an expanding number of markers to be used in developing increasingly detailed characterization of brain tumor cellular interactomes.

### Conclusion.

There are major differences in the immune landscape of CNS tumors that likely influence immune effector functions. Multispectral imaging has the potential to increase understanding of immune cell distribution in different parts of brain TMEs and, in so doing, provide information regarding the basis of tumor response to immune therapies.

## Methods

### Tissue processing and orientation.

Upon the completion of the en bloc resection, which in most cases included the surrounding brain parenchyma, the surgeon created a wedge that spans the necrotic core to adjacent brain parenchyma that could be mounted on a slide. This wedge was immediately fixed with 10% formalin and embedded in paraffin. Slides were prepared at 4 μm tissue thickness. Standard orientating H&E slides were generated and segmented by a board-certified neuropathologist into brain-tumor interface/infiltrating edge, tumor, and necrosis (Figure 1A).

### Nanostring analysis.

RNA (200 ng) was isolated from the formalin-fixed, paraffin-embedded tissues from the tumor-segmented areas (brain-tumor interface/infiltrating edge, tumor, and necrosis) and was analyzed using NanoString gene expression profiling. RNA samples were prepared by ligating a specific DNA tag (mRNA-tag) onto the 3′ end of each mature mRNA, and excess tags were removed via restriction enzyme digestion at 37°C. After processing, using the mRNA sample preparation kit (RNeasy FFPE Kit, Qiagen), the entire 10 μL reaction volume containing mRNA and tagged mRNAs was hybridized with a 10 μL reporter CodeSet, 10 μL hybridization buffer, and a 5 μL capture ProbeSet (for a total reaction volume of 35 μL) at 65°C for 16 to 20 hours. Excess probes were removed using 2-step magnetic bead–based purification with an nCounter Prep Station. The specific target molecules were quantified using an nCounter Digital Analyzer by counting the individual fluorescent bar codes and assessing target molecules. The data were collected using the nCounter Digital Analyzer after obtaining images of the immobilized fluorescent reporters in the sample cartridge using a charge-coupled device camera. Cell population frequency was inferred based on the relative mRNA quantity and is automated based on gene expression.

### Multiplex immunofluorescence staining.

Each antibody was validated using conventional immunohistochemistry and monoplex immunofluorescence staining, in conjunction with the corresponding Opal fluorophore and the spectral DAPI counterstain. The monoplexes were tested at 3 different dilutions, starting with the manufacturer-recommended dilution (MRD), and then MRD/2 and MRD/4, with 1 of 100 tyramide to select the optimal concentration to generate the best signal. The signal was then optimized with different tyramide titers. Reproducibility was evaluated using a positive control of each monoplex with DAPI, a DAPI-alone slide, the negative controls (including 1 unstained slide per tumor type for autofluorescence compensation), tyramide only (treated with hydrogen peroxide quenching solution for endogenous peroxidase masking), and the secondary antibody plus tyramide slides to validate the antibody blocking and nonspecific background. The following antibodies were used in the multiplex analysis: CD3 (Dako Agilent, clone F7.2.38) 1:50 dilution with pH 6 Ag retrieval buffer (ARB), associated with Tyr480 1:350 dilution; p-STAT3 (Cell Signaling, Tyr705 D3A7 XP) 1:200 pH 6 ARB with Tyr520 1:150; CD68 (Agilent, PG-M1) 1:50 pH 9 ARB with Tyr570 1:150; GFAP (Abcam, EPR1034Y) 1:250 pH 6 ARB with Tyr620 1:300; CD163 (Abcam, EPR19518) 1:600 pH 9 ARB with Tyr690 1:100; and CD11c (Abcam, EP1347Y) 1:300 pH 9 ARB with Tyr780 1:100. For metastasis samples, the GFAP antibody was replaced by the pan-cytokeratin (Abcam AE1/AE2) 1:50 pH 9 ARB with Tyr620 1:300. Notably, the GFAP antibody also stains normal astrocytes (66, 67) present in the brain adjacent to the tumor. They were differentiated from tumor cells based on cell morphology.

### Image acquisition and analysis.

Slides were scanned with the Vectra Polaris imaging system (Akoya Biosciences) following the manual’s instructions, with high-power field scan (×40) using the fluorescent mode. The microscope captured the multispectral fluorescent spectra separately at the corresponding tyramide Opal fluorophore wavelength, with preset exposure times, and then these captures were stacked in 1 image (QPTiff) without disrupting the unique fluorescent spectral signature of the markers. The QPTiff image was analyzed in Visiopharm software for the 3 regions of interest: necrosis, tumor, and brain-tumor interface/infiltrating edge.

### Tuning strategies for cellular identification and phenotyping.

All digitized images were analyzed using the Visiopharm software platform. Regions of interest (brain-tumor interface/infiltrating edge, tumor, and necrosis) were identified by board-certified neuropathologists and manually transferred to the Visiopharm platform. A series of custom algorithms were developed for exclusion of red blood cells, nuclei detection, and phenotyping individual cells. For identifying and excluding regions of excessive bleeding, we trained a deep-learning classifier (Deep Lab v3^+^ architecture; input size = 512 × 512 pixels; mini-batch size = 2; learning rate = 1 × 10^-5^; 2500 iterations) using the Opal 480 (Akoya Biosciences), DAPI, and autofluorescence channels to automatically identify these areas and exclude them from further analysis. Training of the convolutional neural network continued until the error rate converged to less than 2%. Magnification used for this task was ×0.5. For detecting nuclei, a pretrained deep-learning algorithm available with the Visiopharm platform (U-Net architecture) was used. The convolutional neural network was trained to identify 3 components of the fluorescent images: (a) DAPI^+^ nuclei; (b) boundaries of DAPI^+^ nuclei; and (c) background. The algorithm magnification was set to ×20 to maximize the ability to capture details in the images. Once nuclei in the sample were identified, the nuclear labels were expanded by 3 μm in all directions to approximate the boundaries of cells, not just DAPI nuclei. Finally, object labels with an area of less than 11 square microns (corresponding to a radius of approximately 2 μm) were removed from further analysis. The cell segmentation was confirmed via visual inspection conducted by trained personnel.

For phenotyping cells, a targeted approach to generate the specific list of phenotypes (i.e., biomarker combinations) was used. Specifically, we were interested in finding phenotypes that were positive for a single biomarker (e.g., CD3^+^, CD11c^+^, CD68^+^, or CD163^+^), double positive for 2 biomarkers (e.g., CD11c^+^CD163^+^, CD68^+^CD163^+^, or CD11c^+^CD68^+^), and triple positive (e.g., CD11c^+^CD68^+^CD163^+^) as well as whether the cell was in an immunosuppressive state (e.g., p-STAT3^+^ or p-STAT3^–^). For a given cell, the classification of each biomarker was gated using 2 independently controlled parameters: signal intensity and percent coverage. During the design of the generalized classification algorithm, classification parameters were iteratively adjusted to maximize accuracy and minimize the occurrence of false positives and false negatives for each biomarker. Biomarker classifications were visually inspected and confirmed by multiple researchers. Once the parameters for accurate classification were optimized, those settings were applied to all images. Once the algorithms were applied to the images, a list of output variables, including counts of each identified phenotype per region (e.g., brain-tumor interface/infiltrating edge, tumor, and necrosis), their fractional contribution to the population within each tissue compartment, their density, and the spatial location in Cartesian coordinates (e.g., center *x* and center *y* coordinates) for each cell on the whole-slide image, were generated.

### G-function and spatial analysis.

The G-function gives the probability of having at least 1 class 2 cell within a R-pixel distance of a class 1 cell (where R = distance); this is mathematically expressed as: *G_x,y_* (*r*) = 1 – e^–λ*yπr*2^, where subscript *x* and *y* indicate the spatial distribution of cell type *y* relative to the cell type *x* being computed; *r* refers to the distance from the reference cell type; and *λ*_γ_ is the overall density of cell type *y* on the slide (68). The AUC of the G-function, for a given distance, allows for the quantification of the degree of infiltration or “mixing” of 1 cell type around another. These AUCs were computed for all dyad interactions. Maximum radius was set at 50 μm, and G-function AUCs were measured at a radius of 15 μm for all dyad interactions. Some of the AUC values were computed to be 0, arising from a very low number/frequency of cells with the specific marker combinations in the different regions (Supplemental Figure 2). Any G-function above 0 at 15 μm in any sample was dichotomized as a positive interaction score.

### Cross pair count analysis.

To analyze the cluster interactions, a framework was used that involved quantifying the number of cells of interest of each type (nonreference) present within 15 μm of each of the reference cells. After defining the reference and nonreference cells, an observation window was created based on the convex hull determined from the *x*, *y* cell coordinates. These coordinates were converted into spatial point patterns before computing the closest pair of points on each pair of cell phenotype point patterns at our distance of interest (*r* = 15). The final required cell counts were computed by consolidating the counts of all nonreference cells queried around a given reference cell. The implementation of this workflow was done using the *spatstat* package in R (R Core Team) (69, 70).

### Gene ontology analysis.

To identify the phenotypes and potential functionality of the different myeloid cell populations, transcriptomic data analysis of the CD45^+^ immune population in GBM (*n* = 7) was done. Differential expression and gene ontology (GO) enrichment analyses were performed in R (version 4.0.3). Raw count data from the Brain Immune Atlas “Human GBM Newly diagnosed: full” scRNA-Seq data set (36) were downloaded and processed using the associated script_scRNA-Seq.R processing script (https://github.com/Movahedilab/Glioblastoma). This data set includes a mix of macrophages, monocytes, DCs, T cells, natural killer cells, and B cells and, thus, required no additional filtering to separate malignant from nonmalignant cells. Each cellular subpopulation was determined based on whether the single cells exhibited expression (counts per million > 0) in the genes encoding CD11c (ITGAX), CD68 (CD68), and CD163 (CD163). Differential expression on each cellular subpopulation was performed using the FindMarkers tool in Seurat (version 3.2.3), with 1 cellular subpopulation being compared with all other cells in the data set under default parameters (71). The final set of differentially expressed genes for each subpopulation included all genes that exhibited an average log fold change of more than 0 and an adjusted *P* value of less than 0.05. GO enrichment analyses on each set of differentially expressed genes were then performed using the topGO package’s “classic” algorithm (version 2.42.0). *P* values from this analysis were adjusted using the Benjamini-Hochberg procedure, and significantly enriched GO terms were then defined as those with an adjusted *P* value of less than 0.05 and a higher number of genes than expected.

### Statistics.

For each immune marker, region (e.g., brain-tumor interface/infiltrating edge, tumor, and necrosis), and type (p-STAT3^+^ and total), a 2-sample Wilcoxon’s rank-sum (unpaired) exact test was performed to compare between glioma and metastasis, and *P* values were computed. To compare across areas for each marker, percentages across regions were compared using 1-way repeated-measures ANOVA, with the percentages transformed by log_10_(*x* +1) to improve the normal approximation. To account for multiple comparisons, *P* values were adjusted using the false discovery rate (72). Adjusted *P* values of less than 0.05 were considered significant.

### Study approval.

Under PA16-1090, approved by the institutional review board of The University of Texas MD Anderson Cancer Center, patients were identified with surgically resectable tumors, diagnosed between April 2018 and January 2020, with an intent to undergo en bloc resection. All patients were screened based on a presumptive radiographic diagnosis of CNS glioma or brain metastasis. Prospective informed consent was obtained from all patients. All patients received standard-of-care intraoperative Decadron (4–10 mg/kg). Study patient demographics and clinicopathological findings were collected from the electronic medical record (Table 1).

## Author contributions

ABH provided experimental design and/or implementation. MO, CK, AM, and SDF collected the data. HN, MO, CK, AR, GR, AM, AMS, CH, RV, AS, SNK, FSV, VAA, PG, SDF, JTH, GNF, JPL, DEW, BAF, JKB, and ABH analyzed the data. HN and ABH wrote the manuscript. HN, MO, CK, AR, GR, AM, AMS, CH, RV, AS, SNK, FSV, VAA, PG, SDF, JTH, GNF, JPL, DEW, BAF, CDJ, LCP, MSL, JKB, and ABH read and approved the final version of the manuscript. HN and MO are co–first authors. Authorship order was decided as follows. MO worked on the initial multiplex panel optimization and visual readings of the images. HN analyzed the immune interactome and single cell analysis, generated the figures, and wrote the manuscript.

## Supplementary Material

Supplemental data

## Figures and Tables

**Table 1:**
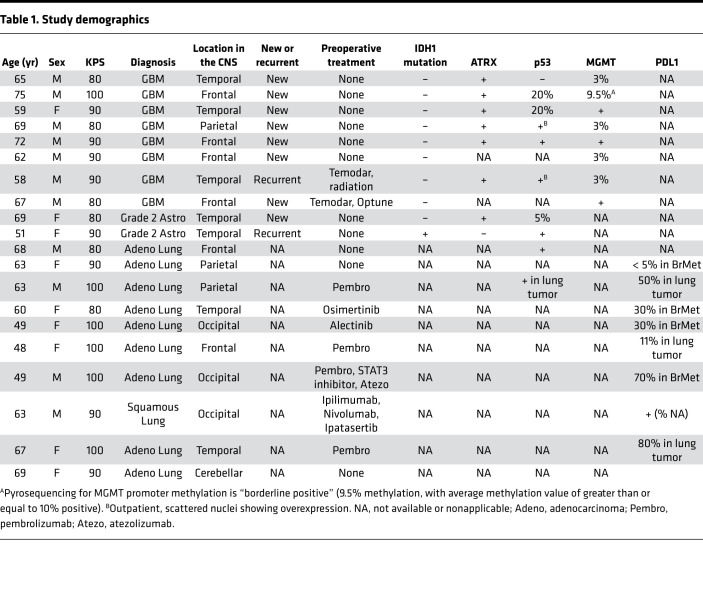
Study demographics

## Version 1 03/22/2022

In-Press Preview

## Version 2 05/09/2022

Electronic publication
