# Supplementary material for: Central nervous system immune interactome is a function of cancer lineage, tumor microenvironment, and STAT3 expression
Source: JCI Insight. 2022 May 9;7(9):e157612. doi: 10.1172/jci.insight.157612 (PMC9090258; doi:10.1172/jci.insight.157612)
Supplement: Supplemental data [file jciinsight-7-157612-s300.pdf]

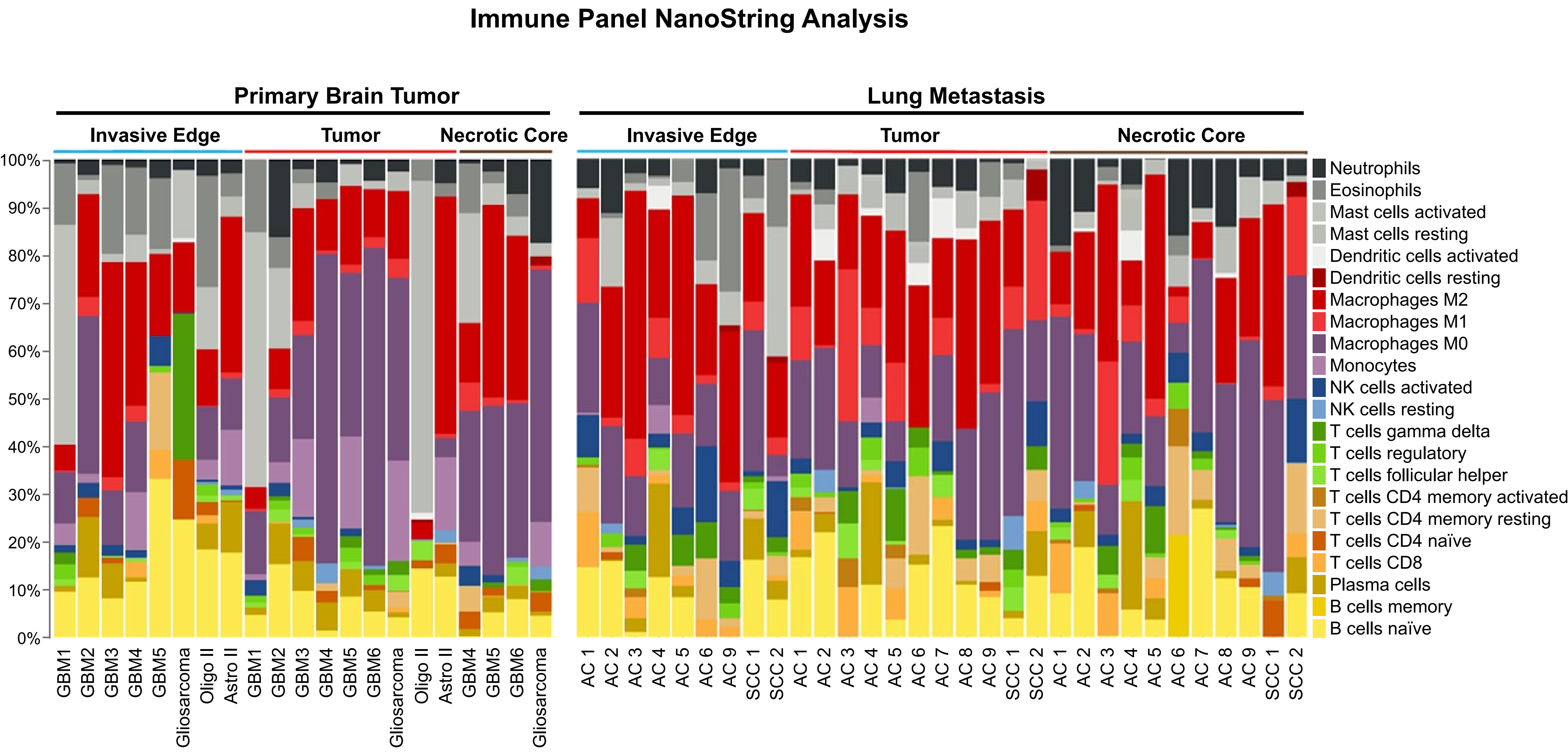

Supplementary Fig. 1

Supplemental Fig. 1: Heat maps showing relative percentage of immune cells in the different regions of GBMs, and lung metastases based on NanoString analysis. Neutrophils are found in the necrotic core and eosinophils at the infiltrating edge in both brain metastases and gliomas. Activated dendritic cells and CD8 T cells are more commonly found in brain metastases and are rarely present in gliomas. The frequency of proinflammatory M1 macrophages predominates in brain metastases relative to gliomas. The necrotic core of GBM and metastases is dominated by M0 and M2 macrophages. M2 macrophages are enriched in the necrotic core and the infiltrating edges in GBM.

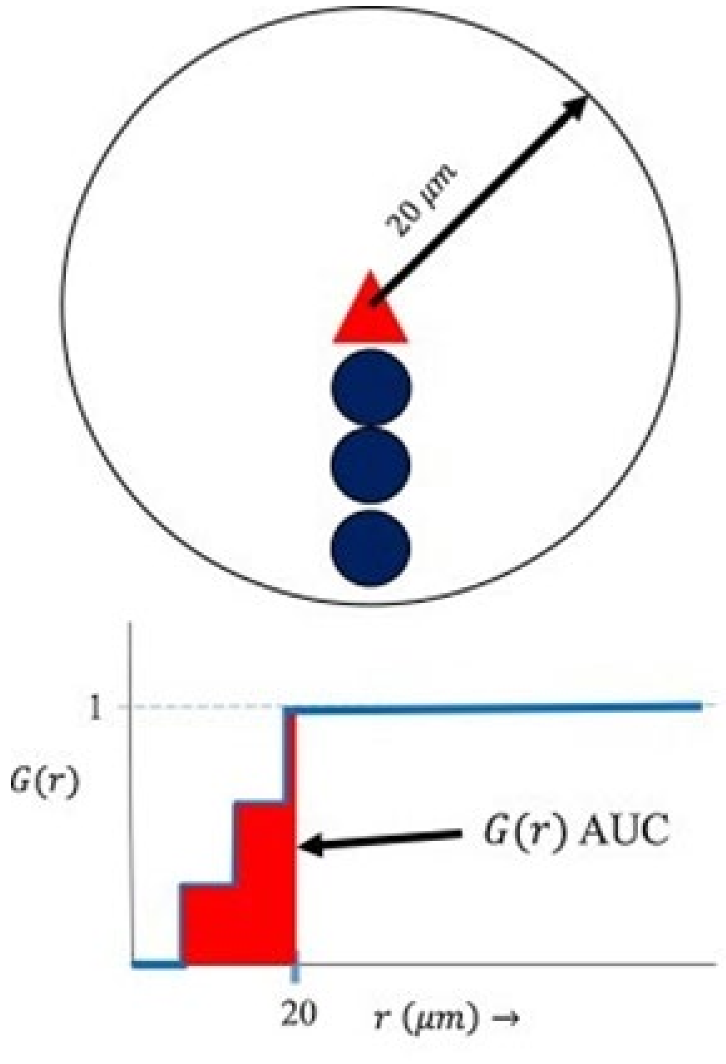

(a) Infiltrative

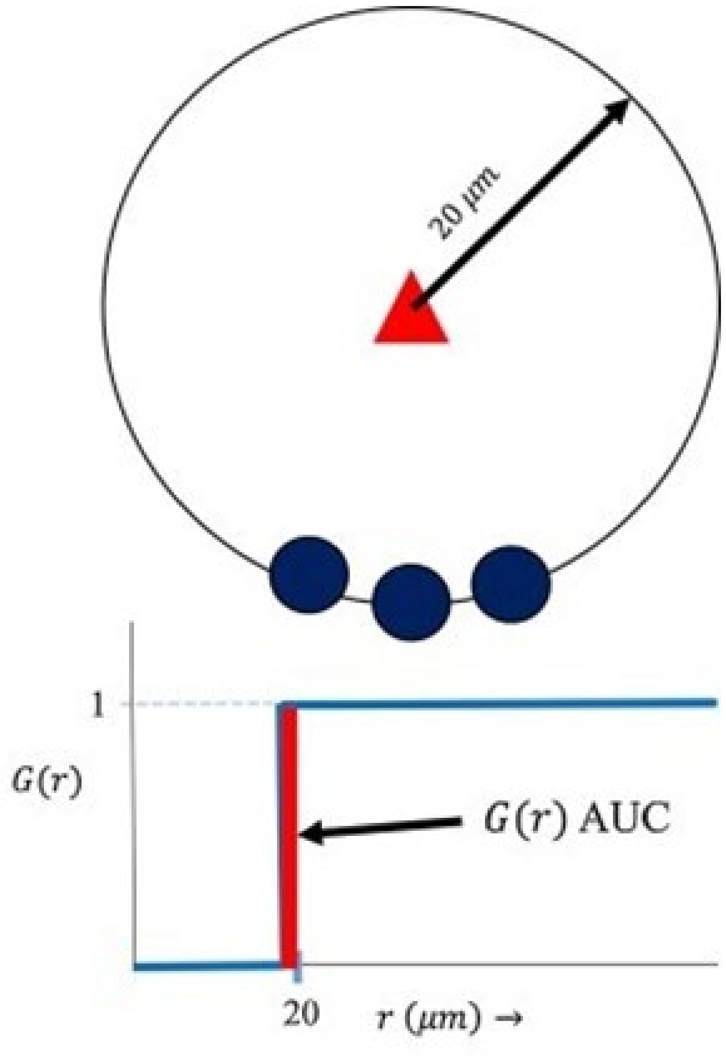

(b) Non-infiltrative

Supplementary Fig. 2

Supplemental Fig. 2: G-Function Analysis. The G-function gives the probability of having at least one cell of Class 2 within R-pixel distance of cell of Class 1 (where R=distance) and is mathematically expressed as: $G_{x,y}(r) = 1 - e^{-\lambda_y \pi r^2}$, where the subscripts 'x' and 'y' indicate that the spatial distribution of cell type 'y' relative to the cell type 'x' is being computed, 'r' refers to the distance from the reference cell type, and λ_y is the overall density of cell type 'y' on the slide (25). Computing the area under the curve (AUC) allows G-function curves to be compared.

Supplementary Table 1: List of the significantly expressed genes in the different myeloid populations and their roles.

CD68+ CELLS (60 Genes)	
**GENE**	**ROLE**
**CD68**	CD68 marker
**RNASET2**	Ribonuclease T2. Abundant in the brain, break down RNA. Is intracellular and secreted within the microenvironment. It is a tumor suppressor gene and favors M1 macrophage polarization (Francesco Acquati, et al. Frontiers Immunology. 2019; Lei Wu, et al. Frontiers Immunology. 2020). Also, hypoxia induce this gene expression and protein secretion in monocyte derived DCs, with regulation of anti-tumor immune response (Sara Monaci et al. International Journal of Molecular sciences. 2021).
**DHRS9**	Dehydrogenase/Reductase 9 expressed in regulatory macrophages (Mregs) (Bitterer Florian et al. 2018; Paloma Riquelme, et al. 2017).
**CX3CR1**	Receptor binds fractalkine for microglia recruitment into the inflamed tissue and can be pro- or anti-inflammatory depending on the microenvironment (Myoungsoo Lee, et al. 2018).
**PLD4**	Phospholipase D family member 4. Expressed in microglia and break down ssDNA. It has a role in regulation of inflammatory cytokine response via degradation of nucleic acids that are TLR9 ligands. Thus prevent TLR9 activation and autoinflammatory processes (Amamda L. Gavin et al. Nature immunology. 2018; Amanda L. Gavin et al. Nature communication. 2021). It is also associated with phagocytosis in activated microglia, favorizing phagocytic clearance by microglia (Yoshinori Otani, et al. Plos One. 2011; Terumasa Chiba, et al. Proceedings of the Japan Academy, Series B, physical and biological sciences. 2016). It has been shown in colon cancer to promote M1 macrophage phenotype (Long Gao, et al. Oncology reports. 2017).
**NAP1L1**	Nucleosome assembly protein 1-like 1. Role in nucleosome assembly, DNA replication and in regulation of cell proliferation (Toshiaki tanaka, et al. 2017).
**IFNGR1**	Interferon gamma Receptor 1 or CD119. Its ligand is INF-γ and is associated with inflammatory immune response in different context including anti-tumor response. Interferon signaling leads to the phosphorylation of the intracellular domain that induce activation of SOCS1 that negatively regulates JAK-STAT pathway. Induce macrophage/microglia activation and induce crosstalk between neurons and microglia (Makoto Tsuda, et al. PNAS. 2009; Vasiliki Panagiotakopoulou, et al. Nature Communication. 2020).
**RGS10**	Regulator of g protein signaling 10 highly expressed in microglia and neurons. It plays a regulatory inhibitory effect on the immune response induced by microglia (Jae-Kyung Lee, and Malu G Tansey. Progress in molecular biology and translational science. 2015; Mohammed Alqinyah, et al. Molecular pharmacology. 2018). It can be also regulator of pro-inflammatory cytokine production by microglia (Mohammed Alqinyah et al. 2017; Mohammed Alqinyah, et al. 2018).
**ITM2B**	Integral membrane protein 2B role in inhibiting AB amyloid protein deposition.

**RAC1**	Rac family small GTPase 1. Regulator of cell cycle, motility, and cell-cell adhesion.
**TMIGD3**	Transmembrane and immunoglobulin domain containing 3: inhibitor of NF-kappa B activity in osteosarcomas (Swathi V. Iyer, et al. 2016).
**TREM2**	Triggering receptor expressed on myeloid cells 2 induce APOE signaling in neurodegenerative diseases ex. Alzheimer's that dysregulate microglia. (Susanne Krasemann et al. 2017). Also expressed by TAMs in various cancers including gliomas and is associated with tumor progression and resistance to anti-PD-1 immunotherapy (Martina Malgora, et al. 2020).
**BHLHE41**	Basic Helix loop helix family member E41 is a transcription factor. Role in regulation of circadian mechanisms and in immune system as inducer of TH2 maturation associated with humoral immune response. Also, associated with regulation of the immune response and maintaining of Tregs. Regulates IL-1β production. (Buka Samten. Cellular and molecular Immunology. 2017) Expressed in tissue-bound macrophages, like microglia, and might have a role in the maintenance of the cell identity and survival (Rene Rauschmeier, et al. 2019).
**CKB**	Creatine kinase Brain form is upregulated in CD8 T cells after TCR activation and linked to the activation of the creatine transporter gene and pathway… (Stefano Di Biase, et al. 2019; Liangliang Ji, et al. Immunity. 2019). Its role in macrophages/microglia is unclear.
**CALM2**	Calmodulin 2 (ca binding protein): role in cell cycle progression and proliferation.<br>Also, induce JAK2/STAT3/HIF-1/VEGFA pathway activation in macrophages in gastric cancer (Ganggang Mu, et al. 2021).
**RPL23**	Ribosomal protein L23.
**GPR34**	G protein coupled receptor 34 is a receptor of the nucleotide P2Y12-like group, highly expressed in microglia (and other cells macrophages DCs…). Regulator of phagocytosis and apoptosis: Its absence showed altered phagocytic activity in microglia (Julia Preissler et al. 2015; Elisabeth Jager, et al. The Journal of immunology. 2016)
**BIN1**	Bridging integrator 1 involved in endocytosis and apoptosis processes.
**PIH1D1**	PIH1 domain containing protein 1 role in ribosomal RNA transcription.
**C12ORF75**	Chromosome 12 open reading frame 75 (prognostic marker in several cancers and associated with immune infiltration of tumors. Guangzhen Cai, et al. 2021). However, role in immunity and glioma is unclear.
**LY86**	Lymphocyte antigen 86 role in innate immune response to lipopolysaccharides and cytokine production. In gliomas role unclear.
**SUSD3**	Sushi domain containing 3. Associated with breast cancer, promoter of estrogen-dependent cell proliferation and regulator of cell-cell and cell-substrate interactions and migration in breast cancer (I. Moy et al. 2015). However, its role in macrophages/microglia within brain tumors is unclear.

**HLA-DMB**	MHC II DM beta chain. It is a chaperone protein that has a role in stabilizing and regulation of the loading of antigens on MHC II in the endosomal compartment (Elizabeth D Mellins, et al. 2013).
**NAA20**	N alpha acetyltransferase 20 (catalytic subunit of the NatB n-terminal-acetyltransferase B) contributes to cell growth and autophagy by regulating AMPK activity. (Taek-yeol Jung et al. 2020).
**FAM105A**	Family with sequence similarity 105 member A. Role?
**CYFIP1**	Cytoplasmic FMR1 interacting Protein 1 regulates membrane ruffles and lamellipodia formation also plays a role in axon outgrowth and in actin filament reorganization (Niels Haan, et al. 2021).
**IGSF6**	Immunoglobulin Superfamily Member 6 expressed especially by macrophages but role unclear.
**APBB1IP**	Amyloid-β precursor protein binding family B member 1 interacting protein. Have a role in actin cytoskeletal remodeling after RAS activation signaling… also is involved in activation and modulation of innate immune response (regulator of recruitment and complement mediated phagocytosis). But also play role in tumorigenesis and tumor progression (Qianyun Ge, et al. journal of cancer. 2021).
**APMAP**	Adipocyte Plasma Membrane Associated Protein is an endogenous inhibitor of Aβ protein production in the brain (Sebastien Mosser, et al. Hum mol genetics. 2015).
**CH25H**	Cholesterol 25-hydroxylase has a role in synthesis of 25-hydroxycholesterol. Expressed by microglia and macrophages. Regulate lipid metabolism and innate immune response. It is increasingly expressed when Toll-like receptors are activated and is an amplifier of immune response against viruses and induce secretion of IL-1β in an APOE isoform dependent manner in Alzheimer's disease (Jin Zhao, et al. Viruses. 2020; Elizabeth S. Gold et al. PNAS. 2014; Man Ying Wong et al. Journal of Neuroinflammation. 2020). In some cancer it's downregulated (Angelica Ortiz, et al. Cancer cell. 2019).
**FOS**	Binds to c-Jun to form activator protein 1 (AP-1), is part of the MAPK/ERK pathway. This latter is a major transcriptional factor and regulator of cells. Inhibition of FOS/AP-1 pathway leads to MAPK/ERK activation and induce IL-10 secretion… (Huynh T. Hop, et al. 2018) (Vasileios Atsaves, et al. 2019). In gliomas expressed with other genes in microglia and may be predictive of microglial clusters 2 that are transcriptionally activated (Natalia Ochoka, et al. 2021).
**EPB41L2**	Erythrocyte membrane protein band 4.1 like 2 has a role in mitosis especially anaphase where it is required for dynein-dynactin complex and NUMA1 recruitment at the mitotic cell cortex.

CSF2RA	Colony stimulating factor 2 receptor subunit alpha or CD116, its Ligand is GM-CSF or CSF2. Highly expressed on innate immune cells within the TME and glioma tumor cells. Cytokine receptor controls production, differentiation, and function of granulocytes and macrophages. Its presence shows immune dependent antitumor effect of GM-CSF, as this latter can have an immune independent effect (Rogio G. Urdinguio et al. Cancer research. 2013; Fengling Chen. Scientific Research publishing. 2021).
**SYNDIG1**	Synapse differentiation inducing 1. Regulate functional excitatory synapse development (Evgenia Kalashnikova et al. Neuron. 2010).
**FSCN1**	Fascin Actin-bundling protein 1 has a role in the organization of actin filament bundles and the formation of micro-spikes, membrane ruffles and stress fibers (Katrin Deinhardt, et al science signaling. 2011). Also, important of cell protrusions formation and for cell migration, adhesion, and invasion (Lin Chen et al. nature 2010; Shengyu Yang et al. Journal of biological chemistry. 2013; Shuisheng Yu, et al. Frontiers in Pharmacology. 2021).
**OLFML3**	Olfactomedin like 3 gene represent one of the specific genes that define microglia and discriminate them from the other macrophages. Codes for a secreted protein that plays a role in the development of the CNS, especially dorsoventral patterning. Induced by TGFB1 that is responsible for microglia activation and maturation (Nicolas Neidert, et al. Frontiers in immunology. 2018).
**TMEM119**	Transmembrane protein 119 is a microglia subset marker (Jun-ichi Satoh, et al. Neuropathology. 2016; Simone Bohnert, et al. International journal of legal medicine. 2020).
**HPGDS**	Hematopoietic prostaglandin D synthase. Catalyzes the conversion of PGH2 to PGD2 that has a wide range of functions. It can be pro- or anti-inflammatory depending on the context (Myungsoo Joo and Ruxana T. Sdikot. Mediators Inflammation. 2012).
**C10ORF54**	Code for VISTA protein (V-domain Ig suppressor of T cell activation) is an immune checkpoint and is highly expressed by TAMs and Tregs (Elizabeth C. Nowak, et al. Immunology Rev. 2018).
**GNG7**	G protein subunit Gamma 7. Modulator and transducer in transmembrane signaling pathways: role in the regulation of adenylyl cyclase signaling in certain regions of the brain.
**SHTN1**	Shootin 1. Role in cytoskeleton organization.
**EVI2B**	Ecotropic viral integration site 2B is a regulator of myeloid cells differentiation (Polina Zjablovskaja, et al. Cell Death and differentiation. 2017). It is associated with higher infiltration of CD8+ T cells in melanoma and is correlated with INF-γ signature genes (Satoru Yonekura and Kosuke Ueda. Cancers. 2021).

LPCAT2	Lysophosphadylcholine Acyltransferase 2 has a role in the storage of phospholipid precursors of PAF and eicosanoids: catalyzes the membrane biogenesis in inflammatory cells while producing PAF (platelet activating factor). It co-localizes with TLR4 and plays a role in macrophage inflammatory expression in response to bacterial ligands stimulation. (W. Abate, et al. Scientific reports. 2020). In several cancers the expression of this gene is associated with poor prognosis and anti-inflammatory response (Juliana L. Souza et al. Frontiers in Oncology. 2020; Alexia Karen Cotte, et al. nature communications. 2018).
P2RY13	The second most highly expressed on microglia after P2RY12, regulates microglia morphology by favorizing processes and ramification development, surveillance, and resting levels of IL-1B release was higher. (Vasiliki Kyrargyri et al. Glia. 2020) and these genes might be downregulated in certain subtype of microglia in glioblastoma microenvironment (Sybren L. N. Maas, et al. Journal of Neuroinflammation. 2020).
CCL3	Chemokine ligand 3 or macrophage inflammatory protein-1alpha (MIP-1α) role in macrophage activation, TNF-α and INF-γ secretion (Daniel Gibaldi, et al. 2020; Antonella Sanguinetti, et al. 2018; Teilo H. Schaller et al. Expert Rev Clin Immunol. 2017).
PTAFR	Platelet activating factor receptor has an immunosuppressive role when activated via COX-2 mediated mechanisms (Jesus A. Ocana, et al. The journal of Immunology. 2018; Ravi P Sahu et al. JSM cell dev biol. 2014).
LPAR5	Lysophosphatidic Acid Receptor 5 stimulate tumor progression and metastasis, and is a pathway used for immunesuppression in the TME (Sue Chin Lee, et al. Cancers. 2020). Also inhibits B cell antigen receptor signaling and antibody response (Jiancheng Hu, et al. Journol of Immunology. 2014). Also suppresses CD8 Tcell cytotoxicity via disruption of TCR signaling (Divij Mathew, et al. Frontiers in Immunology. 2019; Yu-Hsuan Lin, et al. Cells. 2021).
ADORA3	Adenosine A3 Receptor is a sensor of adenosine that is secreted by tumor cells and activate immune suppressive pathways (Van der Putten, et al. Journal of immunology. 2009; Sybren L. N. Maas, et al. Journal of Neuroinflammation. 2020).
LTC4S	Leukotriene C4 Synthase leads to leukotriene C4 production. This latter mediator of inflammation. But in cancer TME it contributes to active tumor growth and resistance to immunotherapy (Wen Tian et al. Frontiers in Pharmacology. 2020).
BLNK	B cell linker protein plays a role in B cell receptor signaling. Its downregulation leads to hematologic cancers like leukemia and lymphoma. (Nupur Gupta et al. Cancer Research. 2008). In solid tumors it might be promoting tumor progression (Shengchi Zhang et al. Canadian Science Publishing. 2021). In Microglia associated with neurodegenerative diseases, it is associated with TREM2 pathway and leads to NFkB and ERK1/2 activation between others (Lorenza Magno, et al. Molecular Neurodegeneration. 2021).
GPN3	GPN-Loop GTPase 3. Its role in transportation of RNA polymerase II into the nucleus and in cellular proliferation (Clement Carre and Ramin Shiekhattar. Mol Cell Biol. 2011; Monica R Calera et al. Biochimica Biophysica Acta. 2011).

CEBPD	CCAAT enhancer binding protein delta is a transcription factor in many biological processes: proliferation, differentiation, motility, and immune response. Can crosstalk with NF-kapa B and induce pro-inflammatory response and activation of macrophages (Chiung-Yuan Ko, et al. 2015).
SOCS6	Suppressor of cytokine signaling 6. Is a tumor suppressor gene in prostate cancer and colorectal cancer (Dongbo Yuan et al. Current Cancer Drug Targets. 2018; E. Letellier, et al. British journal of cancer. 2014). The SOCS family are inhibitors of JAK/STAT pathway (especially SOCs1 and 3). In myeloid cells, SOCS6 negatively regulate insulin signaling through interaction with IRS-4 → insulin metabolism is associated with macrophages polarization process → unclear role of SOCS6 in this mechanism… (Sarah M McCormick and Nicola M Heller. Frontiers in Immunology. 2015).
IER2	Immediate Early Response 2 is a transcription factor has a role in regulation of cellular responses and in regulation of tumor progression and metastasis (A. Neeb, et al. Oncogene. 2012; Lenka Kyjacova, et al. Oncogene. 2021).
CYTL1	Cytokine Like 1 or C17. Is only expressed in CD34+ immune cells and is a secreted protein that resembles IL-8 and is associated with CCR/ERK/Sox9 signaling pathways. It is also similar to IL-2 and its inhibition in certain cancers had led to decrease of tumor proliferation. But its definite role is not clear (Sipin Zhu, et al. Cell Mol Life Sci. 2019).
CCL4L2	CC-motif chemokine ligand 4 like 2. Induces chemotaxis of cells expressing CCR5 and CCR1.
CCL3L3	CC-motif chemokine ligand 3 like 3 or MIP-1α isoform LD78. It is a ligand for CCR1, CCR3 and CCR5.

CD11C+ CELLS (8 Genes)

GENE	ROLE
**ITGAX**	CD11c marker
MALAT1	Metastasis-associated lung adenocarcinoma transcript 1 Or NEAT2 is a non-coding RNA transcript involved in tumor progression and immune suppressive macrophages in various cancers (Swati Mohapatra, et al. 2021).
PRF1	Perforin 1 is usually expressed by T cells and NK cells and is involved in the process of cell destruction and cytotoxicity (Iwona Osinska, et al. Cent Eur J immunology. 2014).
KLRB1	Killer cell lectin like receptor B1 encoding for CD161 surface marker, expressed on myeloid cells, NK, and T cells. Plays an inhibitory role on cytotoxicity, by binding CLEC2D/LLT1 in NK cells and inhibit INF-γ secretion (Christina L. Kirkham and James R. Carlyle. Frontiers in Immunology. 2014). In glioma, can be expressed on myeloid cells and is inhibitory of T cells mediated killing (Nathan D. Mathewson, et al. Cell. 2021).

S100A9	S100 calcium binding protein A9. Role dependent on the concentration secreted, at low concentrations may induce cell growth and proliferation and at higher doses causes inhibition and apoptosis (Siwen Wang, et al. Fontiers immunology. 2018).
SRSF5	Serine and arginine rich splicing factor 5 promote cell proliferation (Yuhan Chen, et al. Nature Communication. 2018).
CD247	T cell surface glycoprotein CD3 Zeta Chain precursor, forms TCR-CD3 complex and has a role in enhancing TCR signaling in the interactions between DCs or APCs with T cells (Wenfeng Ye, et al. Medicine. 2019; P. Wehr, et al. Clin Exp Immunology. 2019; Ombretta Melaiu, et al. Nature communication. 2020).
ACY3	Aminoacylase 3.

CD163+ CELLS (19 Genes)

GENE	ROLE
**CD163**	CD163 marker
FCN1	Ficolin 1: Binds the sugar moieties of pathogen-associated molecular patterns (PAMPs) displayed on microbes and activates the lectin pathway of the complement system and is associated with the inflammatory response in autoimmune diseases (Michihito Katayama et al. international immunology. 2019). It is a monocyte-derived myeloid cell marker and was described in monocyte-DCs type of myeloid cells (Dan Xue et al. Arthritis Rheumatology. 2021). Also, described expressed on MDSCs in hepatocellular carcinoma (Qiming Zhang et al. Cell. 2019).
S100A12<br>S100A8<br>S100A9	Calcium, zinc, and copper-binding protein: play a major role in regulation of inflammatory processes and immune response. It's a TLR4 ligand and RAGE ligand that induces monocyte activation and amplifier of innate immunity (Dirk Foell et al. 2013; Chang Xia et al. 2018). Predictive of anti-tumor immunity and improved survival in certain cancers (Michael Mints et al. 2021), and especially in low grade gliomas S100A family correlated with macrophages, neutrophils, and dendritic cells infiltration (Yu Zhang et al. 2021).<br>S100A8 and A9. Role dependent on the concentration secreted, at low concentrations may induce cell growth and proliferation and at higher doses causes inhibition and apoptosis (Siwen Wang, et al. Fontiers immunology. 2018).
VCAN	Versican protein (proteoglycan) component of the extracellular matrix interacts and is produced by innate immune cells promoting myeloid cell anti-inflammatory responses and immune suppression in cancers (Thomas N. Wight et al. 2014; Mary Y. Chang et al. 2017; Thomas N. Wight, et al 2020).
EREG	Epiregulin belongs to EGF family which binds to EGFR to regulate immune response (David J Riese, et al. Semin Cell Dev Biol. 2014).
IL1R2	IL-1 receptor 2 is a negative regulator of the Il-1 system signaling (Martina Molgora et al. 2018).

**CFP**	Complement Factor Properdin. Secreted by myeloid cells and associated with better tumor infiltration with immune cells and better survival (Alessandro Mangogna et al. Frontiers immunology. 2020).
**CLEC4E**	C-type lectin domain family 4 member E, expressed on myeloid cells has a role in cell sensing of pathogens and damaged cells. Promote tumor progression (Emmanuel C. Patin, et al. 2017).
**THBS1**	Thrombospondin 1 is an adhesive glycoprotein. Has a role in Cell-Cell and Cell-matrix interaction. Also, plays a role in ER stress and is associated with immune response in GBM (Chunxiao Qi, et al. Oncology letters. 2020).<br>However, note that when expressed on glioma tumor cells especially higher grades and is associated with tumor growth and TGFB1 signaling (Thomas Daubon, et al. Nature communication. 2019; Chunxiao Qi, et al. Oncology letters. 2020).
**F13A1**	Coagulation factor XIII A chain. In lung cancer a certain subtype, is associated with high infiltrate of monocytes expressing F13A and associated with poor prognosis (Alessadro Porrello, et al. Nature communication. 2018). Also, associated with M2 macrophages in Renal carcinoma (Yutao Wang, et al. Frontiers genetics. 2021; natalia Ochocka et al. BioRxiv. 2020).
**TYMP**	Thymidine phosphorylase (previously known as ECGF1) expressed in macrophages and promotes angiogenesis within the TME (Akihiko Kawahara, et al. Oncol Rep. 2010; Wei Li and Hong Yue. Trends Cardiovascular Medicine. 2018).
**STAB1**	Stabilin 1 or clever-1: scavenger receptor expressed on immunosuppressive monocytes/macrophages (Maija Hollen, et al. British Journal of cancer, 2020).
**AREG**	Amphiregulin: immune suppression and tumor progression (Moshit Lindzen, et al. 2021).
**RBPJ**	Recombination Signal Binding Protein for Immunoglobulin Kapa J Region. It is a transcription factor, associated with the Notch pathway and in M2 macrophage polarization… (Julia Foldi, et al. Protein Cell. 2016). However, in other papers it states that it is associated with pro-inflammatory macrophages by upregulation of IRF8 and toll like receptors signaling (Haixia Xu, et al. Nature Immunology. 2012). It is important for adequate anti-bacterial immune response (Lan Kang, et al. J. Experimental Medicine. 2020).
**PLTP**	Phospholipid transfer Protein role in cholesterol uptake and is highly expressed in macrophages. Mentioned as pro-inflammatory gene (Mariana Reis-Sobreiro, et al. Cells. 2021).
**HLA-DRB5**	Class II MHC. TAMs expressing HLA-DR are immune suppressive and predictive of tumor progression (April E. Mengos, et al. 2019).

CD11C+CD163+ CELLS (30 Genes)

GENE	ROLE
**ITGAX**	CD11c marker
**CD163**	CD163 marker

**FCN1**	Ficolin 1: Binds the sugar moieties of pathogen-associated molecular patterns (PAMPs) displayed on microbes and activates the lectin pathway of the complement system and is associated with the inflammatory response in autoimmune diseases (Michihito Katayama et al. international immunology. 2019). It is a monocyte-derived myeloid cell marker and was described in monocyte-DCs type of myeloid cells (Dan Xue et al. Arthritis Rheumatology. 2021). Also, described expressed on MDSCs in hepatocellular carcinoma (Qiming Zhang et al. Cell. 2019).
**VCAN**	Versican protein (proteoglycan) component of the extracellular matrix interacts and is produced by innate immune cells promoting myeloid cell anti-inflammatory responses and immune suppression in cancers (Thomas N. Wight et al. 2014; Mary Y. Chang et al. 2017; Thomas N. Wight, et al 2020). Expressed on MDSCs in hepatocellular carcinoma (Qiming Zhang et al. Cell. 2019).
**S100A12**	Calcium, zinc, and copper-binding protein: play a major role in regulation of inflammatory processes and immune response. It's a TLR4 ligand and RAGE ligand that induces monocyte activation and amplifier of innate immunity (Dirk Foell et al. 2013; Chang Xia et al. 2018). Predictive of anti-tumor immunity and improved survival in certain cancers (Michael Mints et al. 2021), and especially in low grade gliomas S100A family correlated with macrophages, neutrophils, and dendritic cells infiltration (Yu Zhang et al. 2021).
**CD300E**	Glycoprotein family CD300, member E. it's a surface receptor that negatively regulate T cell activation, via inhibition of STAT1 pathway and downregulation of MHC II on myeloid cells… (Sara Coletta, et al. Scientific reports. 2020). However, others have described it to be expressed on the surface of monocytes and migratory DCs, and to be on the contrary activating marker of immune response and T cell activation (Brckalo T., et al. Eur J Immunology. 2010; Clark GJ, et al. J Immunotherapy. 2007).
**IL1R2**	IL-1 receptor 2: negative regulator of the Il-1 system signaling (Martina Molgora et al. 2018).
**CFP**	Complement Factor Properdin. Secreted by myeloid cells and associated with better tumor infiltration with immune cells and better survival (Alessandro Mangogna et al. Frontiers immunology. 2020).
**EREG**	Epiregulin belongs to EGF family which binds to EGFR to regulate immune response. (David J Riese, et al. Semin Cell Dev Biol. 2014). Described as a monocyte-derived myeloid cell marker (Dan Xue et al. Arthritis Rheumatology. 2021).
**NAMPT**	Nicotinamide Phospho-ribosyl-transferase is an NAD biosynthetic enzyme is intracellular and can be secreted in the extracellular milieu where it has a role in induction of inflammation, including in cancers. It is associated with inducing INF-γ signaling and STAT1 pathway dependent manner (Valentina Audrito, et al. Frontiers in oncology. 2020; Thomas B. Huffaker, et al. Nature communications. 2021; Hongwei Lv, et al. Cell metabolism. 2021).
**NEAT1**	Nuclear paraspeckle assembly transcript 1 promotes activation of inflammasomes in monocytes/macrophages and thus macrophages activation (Pengfei Zhang et al. 2019), as it can be promotor of cancer progression and metastasis (Gabriel Pisani and Byron Baron. 2020).

**MCEMP1**	Mast cell expressed membrane protein 1, associated with monocytes and mast cells. Has a role in their regulation and differentiation. Is associated with M2 macrophages in gastric cancer and is predictor of poor prognosis (Ting Liu, et al. Research Square. 2021; Daijun Wang et al. Research square. 2021; Gang Hu, et al. Cancer cell int. 2020).
**THBS1**	Thrombospondin 1 is an adhesive glycoprotein. Has a role in Cell-Cell and Cell-matrix interaction. Plays a role in ER stress. And is associated with immune response in GBM (Chunxiao Qi, et al. Oncology letters. 2020).
**LGALS2**	Glycan-binding protein Galectin 2, shown to be associated with better survival in lung carcinoma and a decrease in its expression facilitates tumor growth and metastasis in lung and colon cancers (Marta Uso, et al. Oncoimmunology. 2017; Haiwen Li, et al. Oncogene. 2021). Associated with M1 macrophage polarization and pro-inflammatory function of myeloid cells (Ada G. Blidner, et al. FEBS Letters. 2015).
**CXCL2**	CXC motif Chemokine Ligand 2. This chemokine is associated with angiogenesis, drug resistance and tumor growth in several cancers including brain cancers. It shares the same receptor as IL-8. CXCL2/CXCR2 is associated with ERK1/2 pathway activation (Fenghua Zhang, et al. Medicine. 2021; Ruth M. Urbantat, et al. Int J Mol Sci. 2021; Quan Zhang et al. Frontiers in Immunology. 2021).<br>Note that CXCL1, CXCL2, CXCL3 are known as powerful neutrophil attractant (Laila A. Al-Alwan, et al. The Journal of Immunology. 2013).
**AREG**	Amphiregulin: immune suppression and tumor progression (Moshit Lindzen, et al. 2021).
**S100A6**	S100 Calcium binding protein A6 (Chang Xia, et al. frontiers Immunol. 2018).
**CSTA**	Cystatin A is an acid cysteine protease inhibitor and epidermal SH-protease inhibitor, expressed in epidermal cells and dendritic cells between others. It has a role in immune response against pathogens. In cancers is associated with better survival and patient outcomes but pathogenesis is unclear (Spela Magister and Janko Kos. J cancer. 2013).
**ASGR1**	Asialoglycoprotein Receptor 1 (J. Kenneth Hoober. Int J Mol Sci. 2020).
**MXD1**	Max Dimerization Protein 1 compete with MYC for binding to MAX → transcriptional repressor and a tumor suppressor gene. Also, allows DC maturation and differentiation (David A. Anderson III, et al. PNAS. 2020).
**LYZ**	Lysozyme. Important for defense against pathogens but associated in cancers with modulation of TNF-α/IL-1β pathway and with immune suppressive tumor associated monocyte derived cells (Alberta Bergamo, et al. Int J Mol Sci. 2019; Michael Behring, et al. Cancer Medicine. 2021).

**ADM**	Adrenomedullin is vasodilator, and regulator of apoptosis, migration, proliferation, and differentiation of multiple cell types. Also, it has anti-inflammatory effects. It downregulates TNF-α in macrophages and induce Tregs. It is expressed by macrophages, DCs and T cells. Once expressed on DCs induce downregulation of co-stimulatory markers and reduces their phagocytic functions (Sandrine Rulle, et al. Immunology. 2012).<br>Note that it is Involved in tumor progression and is secreted by tumor cells too (Ignacio M Larrayoz, et al. Journal of translational medicine. 2014; Ramiro Vasquez, et al. frontiers in oncology. 2021).
**CXCL3**	CXC motif Chemokine Ligand 3. It is also a ligand for CXCR1 and CXCR2. Promote cell migration and activation of MAP/ERK pathway. Has a role in induction of angiogenesis (Laila A. Al-Alwan, et al. The Journal of Immunology. 2013).
**SLC2A3**	Solute carrier family 2 member 3 is a facilitated glucose transporter or GLUT3. It is well known to be expressed in neurons. It has been shown to be associated with STAT3 signaling and immunosuppressive macrophages within the TME (Xingxing Yao, et al. Cancer cell international. 2020; Huabin Gao, et al. Fontiers in oncology. 2021).
**TYMP**	Thymidine phosphorylase (previously known as ECGF1) expressed in macrophages and promotes angiogenesis within the TME (Akihiko Kawahara, et al. Oncol Rep. 2010; Wei Li and Hong Yue. Trends Cardiovascular Medicine. 2018).
**LILRA5**	Leukocyte Immunoglobulin Like Receptor A5. Induces pro-inflammatory cytokine secretion when expressed on monocytes/macrophages and at the same time regulate inflammation by induction of IL-10 production (Ainslie Mitchell, et al. European Journal of Immunology. 2008).
**VIM**	Vimentin is an intermediate filament and part of cell cytoskeleton. And has a role in apoptosis and immune response in sepsis when expressed in lymphocytes (Longxiang Su, et al. Scientific reports. 2019).
**RETN**	A secreted protein: Resistin. It promotes adhesion, infiltration, and migration of monocytes, neutrophils and CD4 T cells. Between its receptors is TLR4 (Yanran Li, et al. Frontiers immunology. 2021).
**PLAUR**	Plasminogen activator, urokinase receptor. Associated with immune suppressive TAMs in glioma (Fan Zeng, et al. 2021).

CD11C+CD68+ CELLS (89 Genes)

GENE	Role
**CD68**	CD68 marker
**ITGAX**	CD11c marker
**DHRS9**	Dehydrogenase/reductase 9. Specific and stable marker of regulatory macrophages (Mregs) (Paloma Riquelme, et al. Transplantation. 2017).

**TREM2**	Triggering receptor expressed on myeloid cells 2 induce APOE signaling in neurodegenerative diseases ex. Alzheimer's that dysregulate microglia. (Susanne Krasemann et al. 2017). Also expressed by TAMs in various cancers including gliomas and is associated with tumor progression and resistance to anti-PD-1 immunotherapy (Martina Malgora, et al. 2020).
**RNASET2**	Ribonuclease T2. Abundant in the brain, break down RNA. Is intracellular and secreted within the microenvironment… is a tumor suppressor gene and a potential macrophage-mediate tumor suppressor (Francesco Acquati, et al. Frontiers Immunology. 2019; Lei Wu, et al. Frontiers Immunology. 2020). Also, hypoxia induce this gene expression and protein secretion in monocyte derived DCs, with regulation of anti-tumor immune response (Sara Monaci et al. International Journal of Molecular sciences. 2021).
**PLD4**	Phospholipase D family member 4. Expressed in microglia and break down ssDNA. It has a role in regulation of inflammatory cytokine response via degradation of nucleic acids that are TLR9 ligands. Thus prevent TLR9 activation and autoinflammatory processes (Amamda L. Gavin et al. Nature immunology. 2018; Amanda L. Gavin et al. Nature communication. 2021). It is also associated with phagocytosis in activated microglia, favorizing phagocytic clearance by microglia (Yoshinori Otani, et al. Plos One. 2011; Terumasa Chiba, et al. Proceedings of the Japan Academy, Series B, physical and biological sciences. 2016). It has been shown in colon cancer to promote M1 macrophage phenotype (Long Gao, et al. Oncology reports. 2016).
**OLR1**	Oxidized low density lipoprotein receptor 1 or Lecithin type oxidized LDL receptor 1 (LOX-1): role in tumor progression and metastasis (M. Murdocca et al. 2021).
**TMIGD3**	Transmembrane and immunoglobulin domain containing 3. anti-tumor effect when expressed in osteosarcoma by inhibiting PKA-AKT-NF-kappaB pathway (Swathi V Iyer, et al. nature communication. 2016).
**NAP1L1**	Nucleosome Assembly Protein 1 Like 1. Involved in DNA replication and cell proliferation.
**IFNGR1**	Interferon-γ Receptor 1 or CD119. Its ligand is INF-γ and is associated with inflammatory immune response in different context including anti-tumor response. Interferon signaling leads to the phosphorylation of the intracellular domain that induce activation of SOCS1 that negatively regulates JAK-STAT pathway. Induce macrophage/microglia activation and induce crosstalk between neurons and microglia (Makoto Tsuda, et al. PNAS. 2009; Vasiliki Panagiotakopoulou, et al. Nature Communication. 2020).
**CCL3**	Chemokine ligand 3 or macrophage inflammatory protein-1alpha (MIP-1α) role in macrophage activation, TNF-α and INF-γ secretion (Daniel Gibaldi, et al. 2020; Antonella Sanguinetti, et al. 2018).
**CX3CR1**	Is a microglia marker and Receptor that binds fractalkine for microglia recruitment into the inflamed tissue and can be pro- or anti-inflammatory depending on the microenvironment (Myoungsoo Lee, et al. 2018).

**BHLHE41**	Basic Helix loop helix family member E41 is a transcription factor. Role in regulation of circadian mechanisms and immune system as inducer of TH2 maturation associated with humoral immune response. Also, associated with regulation of the immune response and maintaining of Tregs. Regulates IL-1β production. (Buka Samten. Cellular and molecular Immunology. 2017) Expressed in tissue-bound macrophages, like microglia, and might have a role in the maintenance of the cell identity and survival (Rene Rauschmeier, et al. 2019).
**RAC1**	RAS-related C3 botulinum toxin substrate 1 codes for the RHO GTPase involved in modulation of the cytoskeleton. Associated with multiple cell functions like phagocytosis, migration, neuronal polarization, and cellular differentiation (Reijinders M. R. F. et al. Am. J. Hum. Genet. 2017; Rana El Masri and Jerome Delon. Nature reviews immunology. 2021).
**GLDN**	Gliomedin is a transmembrane and secreted protein that mediates Schwann cell-axon interaction and the molecular assembly of the nodes of Ranvier (Jerome J. Devaux. Am J Pathology. 2012; Huijong Han and Petri Kursula. J. Biol Chem. 2015).
**CH25H**	Interferon-induced gene (Cholesterol 25-hydroxylase), role in anti-tumor immune response (Angelica Ortiz, et al. 2019; Zhen Lu, et al. 2021).
**ITM2B**	Integral membrane protein 2B, also known as BRI2, is a protein expressed in all tissues of the body and is associated with inhibition of AB amyloid protein deposition and prevent Alzheimer's disease (Jungsu Kim et al. J Neuroscience. 2008; J Ghiso, et al. Brain Pathol. 2006).
**PADI2**	Peptidyl arginine deiminase 2 role is inducing citrullination in important proteins in the cell signaling pathways. Thus, it has different impacts on the immunity depending on its targeted pathway. In macrophages it favorizes differentiation and pyroptosis, induces caspase 1 and /or 11 activation and cell death by accumulation of inflammasomes (Hui-Chun YU, et al. International Journal of Molecular Science. 2020; Zhenyu Wu, et al. Frontiers in Immunology. 2021).
**SOCS6**	Suppressor of cytokine signaling 6. It is a tumor suppressor gene in prostate cancer and colorectal cancer (Dongbo Yuan et al. Current Cancer Drug Targets. 2018; E. Letellier, et al. British journal of cancer. 2014). The SOCS family are inhibitors of JAK/STAT pathway (especially SOCs1 and 3). In myeloid cells, SOCS6 negatively regulate insulin signaling through interaction with IRS-4 → insulin metabolism is associated with macrophages polarization process → unclear role of SOCS6 in this mechanism (Sarah M McCormick and Nicola M Heller. Frontiers in Immunology. 2015).
**BIN1**	Myc box-dependent-interacting protein 1. It inhibits cell proliferation via myc dependent pathway (Katherine Elliott, et al. Oncogene. 1999). It also regulates amyloid-β production and is associated with Alzheimer's disease (Toji Miyagawa, et al. Hum Mol Genet. 2016). It regulates phagocytosis in macrophages and its absence leads to auto-immune diseases (Gold ES, et al. J Exp Med. 2004; Chang MY. Et al. Cancer Res. 2007).

CKB	Creatine kinase Brain is upregulated in CD8 T cells after TCR activation and linked to the activation of the creatine transporter gene and pathway… (Stefano Di Biase, et al. 2019; Liangliang Ji, et al. Immunity. 2019). Its role in macrophages/microglia is unclear.
LILRA4	Leucocyte immunoglobulin like receptor A4 recognizes the restriction factor Human Bone Marrow Stromal Cell antigen 2 (BST2 or CD137) that is upregulated in cancers. It is described as preferentially expressed plasmacytoid DCs and is related with their differentiation and antigen-presentation (Florence Abdallah, et al. Frontiers Immunology. 2021; Giuseppe Palma, et al. Biochim Biophys Acta. 2012; Katie J Anderson and Rachel L Allen. Immunology. 2009). Others say it's associated with negative regulation of DCs activation (Kouyuki Hirayasu and Hisashi Arase. Journal of Human genetics. 2015).
ID2	Inhibitor of DNA binding 2 is a transcriptional regulator that inhibit E protein transcription factors and mediates CD8 T cell immunity and is associated with memory T cells (Michael A Cannarile, et al. Nature Immunology. 2006). It is also associated with NK cell killing (Erin C Zook, et al. Science Immunology. 2018). Lead to downregulation of FoxP3 and decrease in Tregs (Sung-Min Hwang, et al. Nature Communication. 2018). However, note that when expressed by glioma tumor cells it leads to tumor proliferation and aggressiveness (Cornelia Roschger and Chiara Cabrele. Cell Common Signal. 2017).
CSF2RA	Colony stimulating factor 2 receptor subunit alpha or CD116 is highly expressed by innate immune cells within the TME and in glioma tumor cells too. Its ligand is GM-CSF or CSF2. Cytokine receptor controls production, differentiation, and function of granulocytes and macrophages. Its presence shows immune dependent antitumor effect of GM-CSF, as this latter can have an immune independent effect (Rogio G. Urdinguio et al. Cancer research. 2013; Fengling Chen. Scientific Research publishing. 2021).
FOS	Binds to c-Jun to form activator protein 1 (AP-1), is part of the MAPK/ERK pathway. This latter is a major transcriptional factor and regulator of cells. Inhibition of FOS/AP-1 pathway leads to MAPK/ERK activation and induce IL-10 secretion (Huynh T. Hop, et al. 2018) (Vasileios Atsaves, et al. 2019). In gliomas expressed with other genes in microglia and may be predictive of microglial clusters 2 that are transcriptionally activated (Natalia Ochoka, et al. 2021).
SYNDIG1	Synapse differentiation inducing 1 is included by INF signaling and favorizes excitatory synapse formation (Evgenia Kalashnikova, et al. Neuron. 2010; Inderpreet Kaur, et al. The journal of neuroscience. 2016). Expressed in neurons and microglia (David Gosselin, et al. Science. 2017).
RAMP1	Receptor activity modifying protein 1 is part of the Calcitonin gene-related peptide receptor (CGRP). This signaling has a role in microglia transcriptional activation and regulation of inflammation (Qi An, et al. Journal of Neuroinflammation. 2021; Lila Carniglia, et al. Mediators of Inflammation. 2017).
CYFIP1	Cytoplasmic FMR1 interacting protein 1 has a role in membrane process and lamellipodia formation. Role in axon outgrowth and in actin filament reorganization (Niels Haan, et al. 2021).

**DBI**	Diazepam binding inhibitor binds acyl-CoA esters and is its carrier in the intracellular domain, contributing to lipid metabolism. Also, it is secreted and binds GABA receptors in the extracellular domain. It has a role in psychiatric diseases (Adrien Joseph, et al. Cell Death and Disease. 2020; Adrien Joseph, et al. Cell Death and disease. 2021).
**LINC01235**	Long intergenic non-coding RNA 1235 function in cancer immunity is unclear but showed to be associated with induction of gastric cancer metastasis. (Yu-En Tan et al. Aging. 2020). Also, described to be important in induction of inflammatory response against pathogens (Katharina Walter and Leon N Schulte. RNA Biology. 2021).
**GNG7**	G protein subunit Gamma 7 code for the formation of G protein heterodimer (Subunit 7) that is required for adenylyl cyclase activity. It regulates actin cytoskeleton and inhibit cell division. Also, it is an autophagy inducer by inhibiting MTOR. (Juanjuan Liu, et al. Oncotarget. 2016). Showed as being upregulated in astrocytes upon inflammatory response (Gilles Gasparoni et al. Epigenetics and chromatin. 2018).
**LILRB4**	Leukocyte Immunoglobulin-like receptor subfamily B member 4 is an inhibitor receptor that is associated immune checkpoint pathways. It is expressed on monocytes, macrophages and DCs. It suppresses T cell activation, favorizes Tregs, and Tcell anergy. It inhibits DC-T cell interaction, activate Jak/STAT signaling in macrophages and thus immune suppression (Jianchen Liu, et al. Am J Transl Res. 2020).
**RGS10**	Regulator of g protein signaling 10 highly expressed in microglia and neurons. It plays a regulatory inhibitory effect on the immune response induced by microglia (Jae-Kyung Lee, and Malu G Tansey. Progress in molecular biology and translational science. 2015; Mohammed Alqinyah, et al. Molecular pharmacology. 2018). It is also a regulator of pro-inflammatory cytokine production by microglia (Mohammed Alqinyah et al. 2017; Mohammed Alqinyah, et al. 2018).
**SHTN1**	Shootin 1 has a role in cytoskeleton organization (Shimada T, *et al*. J Cell Biol, 2008; Ergin V, et al. J Mol Biol, 2020).
**SGK1**	Serum/glucocorticoid regulated kinase 1 shown to have an inhibitory role on Tregs and regulate Th17/Treg balance. (Chuan Wu, et al. Cell Rep. 2018). However, in microglia it can be inhibitory, and its disruption induce their activation (Hayato Asai, et al. Int J physiol pathophysiol pharmacol. 2018; Oh-Chan Kwon, et al. EMBO Mol Med. 2021).
**GPR34**	G protein coupled receptor 34 is a receptor of the nucleotide P2Y12-like group, highly expressed in microglia (and other cells macrophages DCs…). Regulator of phagocytosis and apoptosis: Its absence showed altered phagocytic activity in microglia (Julia Preissler et al. 2015; Elisabeth Jager, et al. The Journal of immunology. 2016).
**NAA20**	N-alpha acetyltransferase is the catalytic subunit the N-terminal acetyltransferase B (NatB) complex. Responsible of cellular stability, folding and degradation of most proteins, also their interactions and localization (Dominik Layer, et al. Communication Biology. 2021). Can be associated with cancers but its role in immunity is unclear.

**CCL3L3**	CC-motif chemokine ligand 3 like 3 or MIP-1α isoform LD78.
**PIH1D1**	PIH1 domain-containing protein 1 is part of R2TP complex and is a regulator of RNA transcription (Yuya Kamano, et al. FEBS Lett. 2013; Patrick Von Morgen, et al. Frontiers Genetics. 2015).
**LPAR5**	Lysophosphatidic Acid Receptor 5 stimulate tumor progression and metastasis, and is a pathway used for immunesuppression in the TME (Sue Chin Lee, et al. Cancers. 2020). Also inhibits B cell antigen receptor signaling and antibody response (Jiancheng Hu, et al. Journol of Immunology. 2014). Also suppresses CD8 Tcell cytotoxicity via disruption of TCR signaling (Divij Mathew, et al. Frontiers in Immunology. 2019; Yu-Hsuan Lin, et al. Cells. 2021).
**CEBPD**	CCAAT enhancer binding protein delta is a transcription factor in many biological processes: proliferation, differentiation, motility, and immune response. Can crosstalk with NF-kapa B and induce pro-inflammatory response and activation of macrophages (Chiung-Yuan Ko, et al. 2015).
**C3AR1**	Complement C3a receptor 1 is a receptor for C3a (chemotactic and inflammatory complement) highly expressed on microglia and myeloid cells. It has a role in inducing chemotaxis of inflammatory cells and release of granule enzymes and superoxide anions. However, C3-C3aR activation can induce pSTAT3 expression in microglia similar to IL-6 and induce reactive neurons and astrocytes that favorized Alzheimer's disease (Alexandra Litvinchuk, et al. Neuron. 2018). Also, associated with IL-10 signaling (Jeffrey M Harder, et al. J neuroinflammation. 2020).
**A2M**	Alpha-2-macroglobulin is a powerful broad spectrum protease binding protein (carrier) and protein inhibitor. Is secreted by the liver or locally by macrophages. Binds to LDL receptor related protein 1 (LRP1) and LRP8 in the brain, and has shown to bind pro-inflammatory cytokines, TNF and IL-1α, regulating chronic inflammation. (Atlas of genetics and cytogenetics in oncology: http://atlasgeneticsoncology.org/Genes/GC_A2M.html).
**C12ORF75**	Chromosome 12 open reading frame 75 (prognostic marker in several cancers and associated with immune infiltration of tumors. Guangzhen Cai, et al. 2021). However, role in glioma immune microenvironment is unclear.
**RTN4**	Reticulon 4 role in endoplasmic reticulum remodeling, cell trafficking and apoptosis (Valerio Chiurchiu, et al. Neuromolecular Medicine. 2014). They are associated with leukocyte recruitment at sites of acute or chronic inflammation and inhibition of endothelial cells migration, as well as cytoskeletal remodeling that allow extravasation of the immune cells through the BBB (Acevedo et al. 2004; Yu et al. 2009; Wright et al. 2010; Di Lorenzo et al. 2011; Schanda et al. 2011; Wälchli et al. 2013).
**IGSF6**	Immunoglobulin Superfamily Member 6 expressed especially by macrophages but role unclear (E. E. Bates et al. Immunogenetics. 2000).
**CCL4L2**	CC-motif chemokine ligand 4 like 2. Induces chemotaxis of cells expressing CCR5 and CCR1.

**APBB1IP**	Amyloid-β precursor protein binding family B member 1 interacting protein. Have a role in actin cytoskeletal remodeling after RAS activation signaling… also is involved in activation and modulation of innate immune response (regulator of recruitment and complement mediated phagocytosis). But also play role in tumorigenesis and tumor progression (Qianyun Ge, et al. journal of cancer. 2021).
**FSCN1**	Fascin Actin-bundling protein 1 has a role in the organization of actin filament bundles and the formation of micro-spikes, membrane ruffles and stress fibers (Katrin Deinhardt, et al science signaling. 2011). Also, important of cell protrusions formation and for cell migration, adhesion, and invasion (Lin Chen et al. nature 2010; Shengyu Yang et al. Journal of biological chemistry. 2013; Shuisheng Yu, et al. Frontiers in Pharmacology. 2021).
**IER2**	Immediate Early Response 2 is a transcription factor has a role in regulation of cellular responses and in regulation of tumor progression and metastasis (A. Neeb, et al. Oncogene. 2012; Lenka Kyjacova, et al. Oncogene. 2021).
**EGR1**	Early Growth Receptor 1 associated with inhibition of macrophage activation and pro-inflammatory functions and with tumor progression (S. B. McMahon and J. G. Monroe. 1996; Marco Trizzino, et al. 2021; Bin Wang et al. 2021; Ji Yun Lee, et al. 2021).
**DUSP1**	Dual specificity phosphatase 1 negative regulator of MAPK phosphatase and ERK pathways (Jiliang Shen, et al. 2016).
**OLFML3**	Olfactomedin like 3 gene represent one of the specific genes that define microglia and discriminate them from the other macrophages. Codes for a secreted protein that plays a role in the development of the CNS, especially dorsoventral patterning. Induced by TGFB1 that is responsible for microglia activation and maturation (Nicolas Neidert, et al. Frontiers in immunology. 2018).
**PTAFR**	Platelet activating factor receptor has an immunosuppressive role when activated via COX-2 mediated mechanisms (Jesus A. Ocana, et al. The journal of Immunology. 2018; Ravi P Sahu et al. JSM cell dev biol. 2014).
**GPN3**	GPN-Loop GTPase 3. Its role in transportation of RNA polymerase II into the nucleus and in cellular proliferation (Clement Carre and Ramin Shiekhattar. Mol Cell Biol. 2011; Monica R Calera et al. Biochimica Biophysica Acta. 2011).
**JUNB**	Transcription factor is a regulator of cell cycle, can be an inhibitor of cell proliferation and a tumor suppressor, as it can be a proliferation promotor (Marc Piechaczyk and rosa farras. Biochem Soc Trans. 2008).
**APOC2**	Apolipoprotein C2 associated with innate and adaptive immune response. It has been shown that when this gene is highly expressed it is associated with upregulation of DC maturation and Th1&2 activation pathways, and downregulation of PD-1, PD-L1. It is a secreted protein that interacts with CD36 receptor, that allows interaction between cancer cells and the immune system and CD36 mediate the metabolic adaptation that favorizes Tregs survival and maintenance (Yuqiao Liu, et al. Clinical translational medicine. 2021).

LPCAT2	Lysophosphadylcholine Acyltransferase 2 has a role in the storage of phospholipid precursors of PAF and eicosanoids: catalyzes the membrane biogenesis in inflammatory cells while producing PAF (platelet activating factor). It co-localizes with TLR4 and plays a role in macrophage inflammatory expression in response to bacterial ligands stimulation. (W. Abate, et al. Scientific reports. 2020). In several cancers the expression of this gene is associated with poor prognosis and anti-inflammatory response (Juliana L. Souza et al. Frontiers in Oncology. 2020; Alexia Karen Cotte, et al. nature communications. 2018).
EPB41L2	Erythrocyte Membrane Protein Band 4.1 Like 2. Role?
SUSD3	Sushi domain containing 3. Associated with breast cancer, promoter of estrogen-dependent cell proliferation and regulator of cell-cell and cell-substrate interactions and migration in breast cancer. (I. Moy et al. 2015). Role in macrophages/microglia, brain tumors is unclear.
TMEM119	Transmembrane protein 119 is a microglia subset marker (Jun-ichi Satoh, et al. Neuropathology. 2016; Simone Bohnert, et al. International journal of legal medicine. 2020).
RGS1	Regulator of G protein signaling 1 regulates T cell tumor infiltration and inhibit chemokine and cytokine production by macrophages (Frederic Fercoq and Leo M Carlin. Nature immunology. 2021; Jyoti Patel, et al. Nature communication. 2015). Others have shown the induction of immune suppression by inhibiting RGS1 in neuroinflammatory diseases (Jae-kyung Lee and Josephine Bou Dagher. AAPS. 2016).
CALM2	Calmodulin 2 (ca binding protein): role in cell cycle progression and proliferation.<br>Also, induce JAK2/STAT3/HIF-1/VEGFA pathway activation in macrophages in gastric cancer (Ganggang Mu, et al. 2021).
APMAP	Adipocyte Plasma Membrane Associated Protein is an endogenous inhibitor of Aβ protein production in the brain (Sebastien Mosser, et al. Hum mol genetics. 2015).
HLA-DMB	Class II MHC. Associated with M2 like macrophages (Maurizio Polano, et al. 2021).
CD83	Member of the immunoglobulin family: surface marker of mature dendritic cells and antigen presenting activated immune cells (upregulates MHC II and CD86 on activated APCs and may be expressed on activated Tcells and Tregs (Ziduo Li, et al. 2019; Lina E. Tze, et al. 2011; Xinsheng Ju, et al. 2016).
LILRA2	Leukocyte Immunoglobulin Like Receptor A2 inhibit phagocytosis by myeloid cells and downregulate DC function inhibiting T cell activation. (Hao K. Lu, et al. Plos one. 2012; Delphine J Lee, et al. J Immunol. 2007). Can induce immune response against pathogens and secretion of TNF-α but also promote IL-10 production by macrophages (Florence Abdallah, et al. Frontiers Immunology. 2021).
HAVCR2	Hepatitis A Virus Cellular Receptor 2 code for Tim-3 which is an immune checkpoint that regulate macrophage activation and inhibit Th1 cells induction and T cell activation (Laurent Monney, et al. Nature. 2002; Alberto Sanchez-fueyo, et al. nature Immunol. 2003).

**AXL**	AXL receptor tyrosine kinase is an inhibitor of the innate immune response and favorize immune suppressive phenotype of TAMs and cancer progression (Chenjing Zhu, et al. Molecular cancer. 2019).
**SERPINE1**	Code for a protein plasminogen activator inhibitor 1 (PAI-1) role in hemostasis and coagulation.
**SDCCAG8**	SHH signaling and ciliogenesis regulator is a centrosome associated protein, has a role in mitosis (Yuanmin He, et al. Cancer Science. 2021).
**CD9**	Tetraspanin CD9 or Tspan 29 (CD151) regulates cell adhesion and extravasation and T cell activation at the immune synapse (upregulation of adhesion molecules, increase integrin-dependent ERK-1/2 signaling, and stabilize the TCR-MHC complex) (Raquel Reyes et al. 2018).
**BLNK**	B cell linker protein plays a role in B cell receptor signaling. Its downregulation leads to hematologic cancers like leukemia and lymphoma. (Nupur Gupta et al. Cancer Research. 2008). In solid tumors it might be promoting tumor progression (Shengchi Zhang et al. Canadian Science Publishing. 2021). In Microglia associated with neurodegenerative diseases, it is associated with TREM2 pathway and leads to NFkB and ERK1/2 activation between others (Lorenza Magno, et al. Molecular Neurodegeneration. 2021).
**CCL4**	Also, macrophage inflammatory protein-1beta (MIP-1β) is a proinflammatory chemokine. Induces chemotaxis of cells expressing CCR5 and CCR1. But within the TME dependent on the situation can be suppressive or pro-inflammatory (Naofumi mukaida, et al. Adv Exp Med Biol. 2020).
**BTG2**	B-cell Translocation Gene 2 is an anti-proliferative gene that inhibit differentiation and proliferation (Erik R Abels, et al. Cell reports. 2019).
**MT-ND2**	Mitochondrially encoded NADH dehydrogenase 2. Role?
**PLK3**	Polo like kinase 3 regulator of cell cycle (C Helmke, et al. Oncogene. 2015).
**LIPA**	Lipase A (Lysosomal acid lipase) role in lipid metabolism.
**CEBPB**	CCAAT enhancer binding protein delta is a transcription factor in many biological processes: proliferation, differentiation, motility, and immune response. Can crosstalk with NF-kapa B and induce pro-inflammatory response and activation of macrophages (Chiung-Yuan Ko, et al. 2015).
**P2RY13**	The second most highly expressed on microglia after P2RY12, regulates microglia morphology by favorizing processes and ramification development, surveillance, and resting levels of IL-1B release was higher. (Vasiliki Kyrargyri et al. Glia. 2020) and these genes might be downregulated in certain subtype of microglia in glioblastoma microenvironment (Sybren L. N. Maas, et al. Journal of Neuroinflammation. 2020).

**EGR2**	Early growth response 2 is a transcription factor induces SOCS1 and SOCS3 production that are inhibitors of STAT3. (Suling Li, et al. Immunity. 2012). Regulates T cell activation for optimal response. (Tizong Miao, et al. J Exp medicine. 2017), it positively regulates INF-γ production (Rujuan Dai, et al. BMC immunology. 2020). In macrophages it is related with positive regulation of activation (Tatyana Veremeyko, et al. Frontiers Immunology. 2018).
**RIN2**	Ras and Rab interactor 2 have a role in cell trafficking and endocytosis (Reyhaneh Kameli, et al. Eur J Med Genet. 2020).
**NFKBIA**	NFKB inhibitor alpha encode for the alpha subunit of the IKK protein complex that regulates the transcription factor NFK-B (Pablo Costo-segura, et al. Biomed Res int. 2019).
**KLF6**	Kruppel like factor 6 induce NFK-B inflammatory activation but at the same time can be anti-inflammatory depending on the situations (Saiful E. Syafruddin, et al. Biomolecules. 2020; W A Goodman, et al. Mucosal Immunology. 2016).
**RGS16**	Regulator of G protein signaling 16 is ambiguous can induce inhibition of monocytes activation (J Suurvali, et al. Scand J Immunol. 2015; Jae-Kyung Lee and Josephine Bou Dagher. AAPS journal. 2016).
**RGCC**	Regulator of cell cycle.

CD11C+CD68+CD163+ CELLS (123 Genes)

GENE	ROLE
**CD68**	CD68 marker
**CD163**	CD163 marker
**ITGAX**	CD11c marker
**FTH1**	Ferritin heavy chain 1. Role in storage and detoxification of exogeneous iron in macrophages, preventing cellular oxidative stress and cell death. (Goncalo Mesquita et al. 2020) It is downregulated in most cancers (Zhang-Wei Hu, et al. 2021).
**PLAUR**	Plasminogen activator, urokinase receptor. Associated with immune suppressive TAMs in glioma (Fan Zeng, et al. 2021).
**NAMPT**	Nicotinamide Phospho-ribosyl-transferase is an NAD biosynthetic enzyme is intracellular and can be secreted in the extracellular milieu where it has a role in induction of inflammation, including in cancers… it is associated with inducing INF-γ signaling and STAT1 pathway dependent manner (Valentina Audrito, et al. Frontiers in oncology. 2020; Thomas B. Huffaker, et al. Nature communications. 2021; Hongwei Lv, et al. Cell metabolism. 2021).
**FTL**	Ferritin light chain upregulated in several cancers including gliomas and is correlated with TAMs and Tregs (Zhang-Wei Hu, et al. 2021).
**C5AR1**	Complement C5a receptor 1 or CD88 through binding of C5a induce myeloid-derived suppressor cells and suppresses CD8+ effector T cells and induce Foxp3 expression (Bashir Lawal, et al. 2021).

**TGFBI**	TGF-Betta induced, secreted protein that has a role in immune suppression within the TME (Mark A. Travis and Dean Sheppard. Annual Review Immunology. 2014; Yun Chen, et al. Frontiers Molecular Bioscience. 2021).
**SAT1**	Spermidine/spermine N1-Acetyltransferase 1 regulate polyamine levels in the cell by regulating its transport.
**ACSL1**	Acyl-CoA synthetase long chain member 1 is upregulated in activated pro-inflammatory macrophages in glioma TME and related to immune cell proliferation activation and migration (Melanie Rose, et al. Cancer gene Therapy. 2021).
**HBEGF**	Heparin Binding EGF Like Growth Factor associated with tumor growth, angiogenesis and immune suppressive macrophages that secrete IL-10 (Justin P. Edwards, et al. Journal of Immunology. 2009).
**GLUL**	Glutamate ammonia ligase or glutamine synthetase highly expressed in TAMs related to glutamate metabolism (transforms glutamate into glutamine) and upregulated due to the high secretion of glutamate in the TME by glioma tumor cells. Glutamate is neurotoxic and triggers seizures and glutamine is used to fuel TAMs and tumor cells as source of energy (Judy Choi, et al. Cancer Biol Ther. 2015; Ilio Vitale, et al. Cell Metabolism. 2019).
**SLC11A1**	Iron transporter. Solute carrier family 11 member 1 codes for natural resistance associated macrophage protein 1 induce pro-inflammatory response in microglia/macrophages (Goajian Zhuang et al. 2020).
**LYZ**	Lysozyme. Important for defense against pathogens but associated in cancers with modulation of TNF-α/IL-1β pathway and with immune suppressive tumor associated monocyte derived cells (Alberta Bergamo, et al. Int J Mol Sci. 2019; Michael Behring, et al. Cancer Medicine. 2021).
**HMOX1**	Heme Oxygenase 1 expressed on M2 like macrophages within the TME and has a role in the anti-inflammatory functions of TAMs. Its inhibition leads to Cytotoxic T cell activation and proliferation (Emmanuelle Alaluf, et al. JCI insight. 2020). It is related with poor prognosis and survival in glioma patients (Wenrui Ye, et al. Frontiers cell Dev. Biol. 2021).
**MSR1**	Macrophage Scavenger receptor 1 is an important phagocytic receptor of dead cells, debris, and lipoproteins. It's expression in macrophages leads to activation of JNK signaling that shifts anti-inflammatory macrophages to pro-inflammatory. (Mamman Guo, et al. EMBO journal. 2019). Associated also with anti-metastasis effect and prolonged overall survival in osteosarcoma (Zhiyu Chen, et al. Font. Mol. Bioscience. 2020) and anti-tumor effect in glioma (Hanwen Zhang, et al. Oncotarget. 2016).
**ATP6V0C**	ATPase H+ Transporting V0 Subunit C related with autophagy, intra-cellular vacuoles, and lysosomes, but its role in macrophages within the TME is unclear.

**IFITM3**	Interferon Induced Transmembrane Protein 3 has an important role in inflammation and antiviral responses. Mainly Induced by INRs secretion. It's expression and role in immune cells within the TME is unclear, especially that it's induced by INRs that are known to lead an anti-tumor response. Some have shown it to be upregulating antigen presentation on immune cells by upregulating MHC I and II (Gomez-Herranz M, et al. Cell Signal. 2019; Yanez DC, et al. Immunology. 2020; Shen C, et al. Mol Med Rep. 2016), and associated with anti-inflammatory response (Yun Cai, et al. Frontiers immunology. 2021). Others have shown its correlation with TAMs that express CD68 and CD163 markers and with immune checkpoint expression but still its role is unclear… (Li H, et al. Am J Clin Pathol. 2020). In breast cancer a recent study showed a pro-inflammatory role of this gene in the immune TME, and association with better responses to immune therapies (Ushani S. Rajapaksa et al. Frontiers Oncology. 2020).
**GPNMB**	Glycoprotein non-metastatic melanoma protein B described as being expressed on M2 macrophages (Letian Zhou, et al. Cell Immunology. 2017) and can be secreted in the TME where it promotes tumor progression and metastasis (M. Liguori, et al. Cell Mol Immunol. 2021; Marina Saade, et al. Frontiers Immunology. 2021).
**CLEC5A**	C-type lectin domain containing 5A receptor (MDL-1) is associated with M2 macrophages in glioma TME (Luqing Tong, et al. Cancer gene therapy. 2019).
**CTSD**	Cathepsin D are upregulated in TAMs are shown to be associated with modulation of the TME for tumor progression (Ran Afik, et al. JEM, 2016; Vasilena Gocheva et al. Genes Dev. 2010).
**VIM**	Vimentin is an intermediate filament and part of cell cytoskeleton. And has a role in apoptosis and immune response in sepsis when expressed in lymphocytes (Longxiang Su, et al. Scientific reports. 2019).
**DSE**	Dermatan Sulfate Epimerase is a tumor rejection antigen, it produces dermatan sulfate that is an important glycosaminoglycan on the cell surface that plays a role in immune response and pro-inflammatory processes (Pierre Martinez, et al. Glycobiology. 2015) and are deleted or altered in most cancers including in gliomas (Karthikeyan Subbarayan and Barbara Seliger. Curr Cancer Drug Targets. 2019).
**GRINA**	Glutamate Ionotropic Receptor NMDA Type Subunit Associated Protein 1 regulates cell survival.
**HIF1A**	Alpha subunit of transcription factor hypoxia-inducible factor-1 (HIF-1) regulate cell metabolism to resist hypoxia and associated with induction of angiogenesis.
**CTSL**	Cathepsin L are important enzymes in infections for proper antigen presentation on MHC II on the surface of macrophages (Rajeev M Neppal, et al. International Immunology. 2006). However, in cancer it has been described as upregulated in TAMs by which it's secreted and shown to be associated with modulation of the TME that favors tumor progression and metastasis (Ran Afik, et al. JEM, 2016; Vasilena Gocheva et al. Genes Dev. 2010; Samamtha S. Dykes, et al. Oncotarget. 2019). Its inhibition leads to TAMs cell death and increase in oxidative stress that helps tumor eradication (SJ Salpeter, et al. Oncogene. 2015).

**ELL2**	Elongation factor for RNA polymerase II role in transcription, also in Ig production when expressed in plasma B cells (Anthony Ghobrial, et al. J Mucosal Immunol Res. 2019).
**SDS**	Serine Dehydratase has a role in amino acids metabolism especially serine and glycine.
**MAFB**	MAF Basic leucine Zipper (BZIP) transcription factor B have a role in macrophages differentiation and helps distinguish macrophages from dendritic cells (Michito Hamada, et al. Exp Anim. 2020). It is associated with M2 polarization and cholesterol efflux mechanisms (Hwijin Kim. Scientific Reports. 2017; Irina Larionova, et al. Cancers. 2020), and described in CD163+ TAMs (Victor D. Cuevas, et al. The Journal of Immunology. 2017).
**VEGFA**	Vascular endothelial growth factor A associated with TAMs, wound healing processes and angiogenesis within the TME (ILseon Hwang, et al. Journal of translational medicine. 2020; Lucia Lisi, et al. Oncology Reports. 2020).
**GNA13**	G protein subunit Alpha 13 is associated with immune suppression and tumor progression. Induces IL-6, IL-8 and TGF-β production that favors M2 macrophage and Tregs (Suhail Ahmed Kabeer Rasheed, et al. Oncogene. 2021).
**GABARAP**	GABA type A receptor-associated protein is associated with autophagy in macrophages and leads to immune suppression after dead tumor cells are phagocytosed by myeloid cells. Also, it inhibits STING activation induced by INF signaling (Larissa D. Cunha, et al. Cell. 2018). In glioma it is one of the genes that are upregulated within the immune tumor microenvironment and was showed to be correlated with better prognosis and overall survival of patients in this study by yang xu, et al. Frontiers Oncology. 2020.
**RAB20**	Member of the RAS oncogene family is a GTPase that has an important role in phagosome maturation and is induced by INF-γ in macrophages (Akriti Prashar, et al. Frontier Cell Infect Microbiol. 2017). In TME of glioma, role unknown.
**METRNL**	Meteorin like glial cell differentiation regulator is an inflammatory cytokine produced by activated macrophages and is induced by IL-12, TNF-α among others, and inhibited by TGF-β (Irina Ushach, et al. Jrounal of Immunology. 2018). Others have shown also an association of METRNL with STAT3 activation and M2 polarization of macrophages (Gurpreet S. Baht, et al. Nature metabolism. 2020).
**C1ORF162**	Chromosome 1 open reading frame 162 is a protein with unknown function in innate immunity (Yong-Chen Lu, et al. Cancer Immunol Research. 2019).
**PLIN2**	Perilipin 2 is a lipid droplet associated protein in macrophages. It helps these latter to handle the excess fat and prevents foam cell formation (Brittney R. Coats, et al. Cell Reports. 2017; P. Saliba-gustafsson et al. Atherosclerosis. 2018). In breast cancer it is associated with good prognosis and high infiltration of immune cells. However, in colon cancer it has been shown to have a role in chemoresistance and decrease in CD8 T cell infiltration. In glioma it is expressed by the immune population, but role is unclear (Chunliang Shang, et al. Cell Death and Disease. 2020).
**RNF144B**	Ring finger Protein 144B is a E3 ubiquitin-protein ligase has a role in the ubiquitin process and protein degradation. Depending on which TLR signaling it can activate or inhibit NFK-B in macrophages (Suk-Hwan Baek, et al. Journal of Immunology. 2016).

**ATF3**	Activating transcription factor 3 has a role in glucose metabolism, immunity modulation and cancer progression. It can be a pro-inflammatory agent as anti-inflammatory depending on the situations as it can be a tumor suppressor gene or oncogene (Hui-Chen Ku and Ching-Feng Cheng. Fontiers in Endocrinology. 2020). Some have shown that it is associated with M2 polarization of macrophages (Hao Sha, et al. Molecular medicine reports. 2017; Cong He, et al. Fontiers in cell and developmental biology. 2021).
**CTSB**	Cathepsin B like the others Cathepsins it is related with immune suppressive macrophages that favors tumor progression and metastasis (Olga Vasiljeva, et al. Cancer Research. 2006; Ran Afik, et al. JEM. 2016). It is also expressed by microglia within glioma TME that helps tumor progression (Hailong Liu, et al. Cell Reports. 2021).
**MT-ATP8**	Mitochondrial encoded ATP synthase membrane subunit 8 associated with cellular metabolism and ATP synthesis.
**SDCBP**	Syndecan Binding Protein gene coding for Syntenin-1 that is a cellular and secreted protein. It has a role in cell migration, adhesion and promotes metastasis within the extracellular matrix like VCAN (Veronica Ferrucci, et al. iScience. 2021).
**CST3**	Cystatin C is associated with enhanced pro-inflammatory response of macrophages to INF-γ with secretion of NO and TNF-α and higher expression of NFK-B in macrophages (Katarina H Frendeus, et al. International Journal of biochemistry and cell biology. 2009). Also, it is involved with better MHC II antigen presentation, maturation of dendritic cells etc. (Mengting Zi, and Yuekang Xu. Immunology Letters. 2018).
**NME2**	Nucleoside diphosphate kinase B important for energy production and metabolism. Some showed that they might have anti-metastatic capacity when secreted systemically (P. Romani et al. Laboratory Investigation. 2017).
**RHOB**	Ras Homolog family member B plays a role in hypoxia-induced activation of macrophages (Gaoxiang Huang, et al. 2015), and pro-inflammatory cytokine production, part of IL-2 pathway (Shuyuan Liu et al. 2017).
**SOD2**	Superoxide dismutase 2 has a role in the protection of macrophages from oxidative stress and is related with M1 macrophage activation (Caitlin V. Lewis, et al. Journal of immunology research. 2019). Some showed that it's secretion by macrophages might contribute to a favorable microenvironment for tumor cell migration and metastasis (Li Yi, et al. Scientific Reports. 2017). In glioma it was described as a tumor protective gene expressed by microglia/macrophages (Sybren L N Maas, et al. Journal of Neuroinflammation. 2020). However, others showed an upregulation of this gene with M1 phenotypical genes after anti-CD47 treatment in microglia and macrophages within glioma TME (Gregor Hutter, et al. PNAS. 2019).
**MTRNR2L12**	Humanin like 12 protect macrophages from oxidative stress, is antiapoptotic and associated with cellular repair ad growth within the brain (Maria L. Elkjaer, et al. Acta Neuropathologica Communications. 2019). But it's role within the TME is not fully clear.

**ZNF331**	Zinc finger protein 331 is a transcription factor associated with maintaining certain gene enhancers necessary for macrophages identity and function. However, this gene is downregulated in the presence of INF-γ like MAF transcription factor. Actual role in the immune response of macrophages is still unclear (Kyuho Kang, et al. Immunity. 2017).
**NUPR1**	Nuclear Protein 1 is a transcription factor that is associated with ferroptosis inhibition and inhibition of non-apoptotic cell death. This has been shown to promote immune suppression in TAMs and tumor support. (Dai E. et al. Autophagy. 2020; Jiao Liu, et al. Nature Communications. 2021).
**RAB31**	Member RAS oncogene family (Ras-related protein 31) is associated with phagocytosis function enhancement via PI3K/Akt signaling activation (Jeremy C Yeo, et al. Molecular Biology of Cell. 2015).
**MGAT1**	Alpha-1,3-Mannosyl-Glycoprotein 2-β-N-Acetylglucosaminyltransferase increase GLUT1 expression and is shown to be highly expressed in glioma tumor cells and induce tumor survival and progression (Yinan Li, et al. FEBS Letters. 2020). However, in immune cells especially, in monocytes derived cells, this protein is highly expressed and has a role in glycogen production, but also immune suppression (Francois Trottein, et al. Glycoconj Journal. 2009; Marcia S. Pereira, et al. Frontiers in immunology. 2018).
**RPS4Y1**	Ribosomal Protein S4 Y-Linked 1 role in protein synthesis and is used as quality control to differentiate sexes: it is expressed on males (Qi Yang, et al. Frontiers in immunology. 2021; Rogan A. Grant, et al. Nature. 2021).
**LMNA**	Lamin A/C are nuclear proteins and associated with cell motility, differentiation, and structural stability. In macrophages it induces NFK-B expression and pro-inflammatory functions (Niina Dubik and Sabine Mai. Cancers. 2020). However, some have shown that in TAM the expression of Lamin A can reflects the level of SIRPα expressed and the stiffness of the TME due to decrease in phagocytic activity of macrophages (Angela Saez, et al. International Journal of Molecular Sciences. 2020).
**LGALS1**	Galectin 1 associated with immune suppressive macrophages in glioma TME (Qun Chen, et al. International Journal of Cancer. 2019)
**NR4A1**<br>**NR4A2**<br>**NR4A3**	Nuclear receptor subfamily 4 group A member 1 same as NR4A2 and NR4A3 are associated with TGF-β activation, immune suppression, and tumor cells migration within the TME in breast cancer (Daniel Crean and Evelyn P. Murphy. Frontiers in Cell and Developmental Biology. 2021). Also, in other diseases like atherosclerosis, deletion of this gene is associated with pro-inflammatory activation of macrophages (Richard N Hanna, et al. Circulation Research. 2012). Nuclear Receptor Subfamily 4 Group A Member 2 shown to favorize M2 macrophages (Sahil Mahajan, et al. journal of biological chemistry. 2015).
**ZFAND5**	Zink finger AN1-type containing 5 is associated with protein ubiquitination and degradation and its role is unclear (Donghoon Lee, et al. PNAS. 2018).
**TYMP**	Thymidine phosphorylase (previously known as ECGF1) expressed in macrophages and promotes angiogenesis within the TME (Akihiko Kawahara, et al. Oncol Rep. 2010; Wei Li and Hong Yue. Trends Cardiovascular Medicine. 2018).

**MFSD1**	Major Facilitator Superfamily Domain Containing 1 is a lysosomal transporter is predictor of good prognosis if expressed in M2 macrophages in renal cell carcinoma (Yutao Wang, et al. Frontiers in Genetics. 2021). In glioma it is also highly expressed by M2 macrophages (Maurizio Polano, et al. Cells. 2021), but role is unknown.
**VMO1**	Vitelline Membrane Outer Layer 1 Homolog role is unknown in TAMs (Emilie P. Buddingh, et al. Human Cancer Biology. 2011).
**KLF4**	Kruppel-like factor 4 is associated with TGF-β signaling and IL-1β and depending on the situation can be activator or inhibitor of immune response mediated by macrophages (Amr M. Ghaleb and Vincent W. Yank. Gene. 2017).
**NEAT1**	Nuclear paraspeckle assembly transcript 1 promotes activation of inflammasomes in macrophages and thus macrophages activation (Pengfei Zhang et al. 2019), as it can be promotor of cancer progression and metastasis (Gabriel Pisani and Byron Baron. 2020).
**HLA-DRB5**	Class II MHC. TAMs expressing HLA-DR are immune suppressive and predictive of tumor progression (April E. Mengos, et al. 2019).
**S100A10**	S100 calcium binding protein A10 is known as plasminogen receptor and is associated with immune suppressive macrophage infiltration and migration to the TME (O'Connell PA, et al. Blood. 2010; Kyle D Phipps et al. Cancer Research. 2011).
**SOCS3**	Suppressor of cytokine signaling 3 activation leads to inhibition of STAT3 activation (Berit Carow and Martin E Rottenberg. Frontiers in Immunology. 2014) and promotes M1 polarization of macrophages (H Qin, et al. journal of immunology. 2012). Also has an important role in efficient phagocytosis processes (Peter Gordon, et al. Journal of Leukocyte Biology. 2016).
**POLD4**	DNA Polymerase Delta 4 has a role in replication and repair (Qin Miao Huang, et al. Biochemical Biophysical Research Communications. 2010).
**THBD**	Thrombomodulin role in homeostasis, induce protein C activation and thrombin inhibition (yi-Heng Li, et al. Journal of Biomedical Science. 2012). It also has a role in adhesion and extravasation of leukocytes from vasculature to the site of inflammation (Wei-Ling Lin, et al. Immunology and Cell Biology. 2017; Houra Loghmani and Edward M. Conway. Blood. 2018).
**LITAF**	Lipopolysaccharide induced TNF factor is a gene coding for TNF-α secretion by macrophages (Kristen N Bushell, et al. PlosOne. 2011; Junrong Zou, et al. Medicine Reports. 2015).
**ANXA2**	Annexin A2 activate macrophages via TLR4 (Jennifer F. A. Swisher, et al Blood. 2010), but within the TME it is shown to promote M2 macrophages and tumor progression and metastasis (Nunzia Novizio, et al. International Journal of Molecular Sciences. 2021) and is a potential marker of immunosuppression in gliomas (Kaiming Ma, et al. Scientific Reports. 2021).
**PSAP**	Prosaposin protein is a lysosomal hydrolase has a role in protein degradation and has been shown to have anti-metastasis effects (Suming Wang, et al. Science Translational Medicine. 2018) but in other cancer it has the opposite effect (Seiichiro Ishihara, et al. Cancer Research. 2017; Yang Jiang, et al. EBioMedicine. 2018).

**HLA-DQA2**	Shown to be expressed on TAMs in gioblastoma and related to activated microglia and macrophages (Xiateng Cui, et al. Fontiers in Oncology. 2021).
**CXCL16**	Chemokine produced by innate immune cells. CXCR16 receptor is highly expressed by microglia in the brain, and CXCL16 induce microglia polymerization toward an anti-inflammatory phenotype in glioblastoma (Francesca Lepore et al. 2018).
**MTRNR2L8**	Humanin like 8 has been shown to have a role in protecting cells from oxidative stress, hypoxia, etc. (Kelvin Yen, et al. Journal of molecular endocrinology. 2013; Chenghan Lee, et al. Trends Endocrinology Metabolism. 2013).
**SRGN**	Serglycin has a role in granules and vesicles formation and secretion process of bioactive molecules like granzyme B, etc. to the extracellular matrix. It is upregulated in activated macrophages that induce inflammatory response, can be induced by TNF, IL-1β, etc. but within the TME in different contexts this protein secreted by immune cells can help in promoting tumor progression and metastasis (Angeliki Korpetinou, et al. Frontiers in Oncology. 2014).
**ADM**	Adrenomedullin is vasodilator, and regulator of apoptosis, migration, proliferation, and differentiation of multiple cell types. Also, it has anti-inflammatory effects. It downregulates TNF-α in macrophages and induce Tregs. It is expressed by macrophages, DCs and T cells… once expressed on DCs induce downregulation of co-stimulatory markers and reduces their phagocytic functions… (Sandrine Rulle, et al. Immunology. 2012).
**CDKN1A**	Cyclin dependent kinase inhibitor 1A or p21 is a cell cycle regulator (NCBI Gene ID: 1026), and inhibitor of IL-1β signaling in activated macrophages and favors M2 polarization (John C. Scatizzi, et al. European Journal of Immunology. 2009; Biao Ma, et al. Molecular Medicine Reports. 2018).
**MT-ND4L**	Mitochondrially Encoded NADH-Ubiquinone Oxidoreductase Core Subunit 4L that code for NADH dehydrogenase subunit 4L protein. This latter has a role in respiratory mitochondrial process in the cell. It has been shown to have a role in the pro-inflammatory induction of macrophages (Takiguchi H., et al. Scientific reports. 2021; Isabelle Duroux-Richard, et al. Frontiers in Physiology. 2021).
**MAT2A**	Methionine Adenosyl transferase 2A has a role in cellular methionine metabolism and ATP production. In monocytes/macrophages it regulated histone methylation pattern and is associated with a pro-tumorigenic phenotype of macrophages (Yan Zhang, et al. Journal of Immunotherapy of Cancer. 2021; Yasaman Barekatain, et al. Nature Communications 2021).
**RGS2**	Regulator of G Protein Signaling 2 has been associated with induction of angiogenesis within the TME (Kimberly C. Boelte, et al. Plos One. 2011). In breast cancer it has been shown to be tumor suppressive (Ji Hyo Lyu, et al. Journal of Cell Biochemistry. 2015).
**G0S2**	G0/G1 switch gene 2. Cell cycle regulator.
**OSM**	Oncostatin M role in the TME is unclear but recently it has been shown to be associated with JAK-STAT3 pathway signaling in GBM and is especially secreted by microglia (Miao Chen, et al. Cancer Cell International. 2021).

MIF	Macrophage Migration Inhibitory factor is associated with immune suppression within glioma TME and can be potential target for glioma therapy (Michel Mittelbronn, et al. Acta Neuropathology. 2011; Katia Mangano, et al. Oncotarget. 2018; Tyler J. Alban, et al. Fontiers in Immunology. 2020).
IFI6	Interferon Gamma (INF-γ) Inducible Protein 16 is an important factor for macrophage activation and sGAS-STING signaling (K L Jonsson, et al. Nature Communications 2017).
GADD45B	Growth Arrest and DNA Damage Inducible Beta is associated with innate immune cell adhesion, phagocytosis, chemotaxis and reactive oxygen species production (Dominic M Salerno, et al. Journal of Cell Physiology. 2012). In TME, it has been associated with innate immune suppression and T cell inhibition (Daniela Verzella, et al. Cancer Research. 2018).
LGALS3	Galectin 3 has a role in M2 macrophage infiltration within the TME and with Angiogenesis (Weizhen Jia, et al. the American Journal of pathology. 2013; Wang-Ming Hu, et al. Frontiers Medicine. 2020).
FN1	Fibronectin 1 is associated with immune suppressive macrophages (Abdullah A. Tarique, et al. American journal of respiratory cell and molecular biology. 2015; Siamon Gordon and Fernando O Martinez. Immunity. 2010; Stephanie A Amici, et al. Frontiers in Immunology. 2017).
SERPINA1	Serpin family A member 1 encode for α1-antitrypsin that is an important protease inhibitor that is expressed by macrophages. Some have shown a pro-inflammatory role of this protein others related it with M2 macrophages (Guttman O, et al. Frontiers in Immunology. 2016; Ying Li, et al. Annual journal of Medicine. 2021; Lurier EB, et al. Immunobiology. 2017).
HSPH1	Heat Shock Protein Family H (Hsp110) Member 1 secreted by macrophage is associated with IL-1β signaling that induce STAT3 activation, but in acute lung injury this process leads to enhanced pro-inflammatory response (Yafeng Liang, et al. Bioscientific Reports. 2020). In Hepatocellular cancer, also secreted within the TME and is associated with immune modulation and chemo sensitivity (liu-Bo Li, et al. Oncology Letters. 2020). But, in glioma TME its role is unclear.
MT2A<br>MT1X<br>MT1G	Metallothionein 2A expressed in innate immune cells and associated with angiogenesis and promoting tumor progression and metastasis in different cancers (Manfei Si and Jinghe Lang. Journal of Hematology and oncology. 2018).<br><br>Metallothionein 1X is associated with low patient survival and poor prognosis in gliomas (Bernadeta Masiulionyte, et al. Scientific Reports. 2019).<br><br>Metallothionein 1G shown to be associated with suppressed TAMs but actual role within the TME is unclear (Xiateng Cui, et al. Fontiers in Oncology. 2021; Bernadeta Masiulionyte, et al. Scientific Reports. 2019).
SLC16A3	Solute carrier family 16 member 3 or MCT4 is a lactate transporter responsible in lactate transportation across plasma membrane into the extracellular compartment and is associated with immune suppressive macrophages and STAT3 signaling (Xingxing Yao, et al. Cancer Cell International. 2020; Heather L. Caslin, et al. Frontiers in Physiology. 2021).

**APOC1**	Apolipoprotein C1 associated with IL-6 signaling, immune suppression and tumor progression in lung cancer (Hui-Ling Ko, et al. Thoracic Cancer. 2014). In glioma, can be expressed on tumor cells, astrocytes, and innate immune cells but the role in cancer immunity is unclear (Cudaback E, et al. Journal of Neuroinflammation. 2012; Petros Evangelou, et al. Medical molecular morphology. 2019). Also, have been shown to have a supporting role in phagocytic functions of macrophages (Domschke G, et al. Scientific reports. 2018).
**IER3**	Immediate Early Response 3 has a role in inhibition of apoptosis and cell proliferation and survival (QIfeng Zhou, et al. International Journal of Molecular Science. 2017).
**CD83**	Member of the immunoglobulin family: surface marker of mature dendritic cells and antigen presenting activated immune cells (upregulates MHC II and CD86 on activated APCs and may be expressed on activated Tcells and Tregs (Ziduo Li, et al. 2019; Lina E. Tze, et al. 2011; Xinsheng Ju, et al. 2016).
**HSPA1A**	Heat Shock Protein Family A (Hsp70) Member 1A is known for its association with endoplasmic reticulum stress and in inducing anti-inflammatory effect (Di Naso et al. Obesity. 2015; Oh et al. Journal of Biological chemistry. 2012; Urszula Brykczynska, et al. Cell reports. 2020).
**FABP5**	Fatty Acid Binding Protein 5 has been shown to be associated with activated pro-inflammatory form of macrophages in Atherosclerosis (Sherri M Moore, et al. Molecular Immunology. 2015). In breast cancer it has been shown to be associated with immune suppressive TAMs and tumor progression (Jiaqing Hao, et al. Cancer Research. 2018; E. Rao, et al. Oncotarget. 2015).
**SLC2A3**	Solute carrier family 2 member 3 is a facilitated glucose transporter or GLUT3. It is well known to be expressed in neurons. It has been shown to be associated with STAT3 signaling and immunosuppressive macrophages within the TME (Xingxing Yao, et al. Cancer cell international. 2020; Huabin Gao, et al. Fontiers in oncology. 2021).
**BAG3**	BAG family molecular chaperone regulator 3 is known to be immune suppressive and pro-tumorigenic. Anti-BAG3 therapeutics are used in combination with abti-PD-1 to treat pancreatic cancer (Vittoria Iorio, et al. Gut. 2018). It has been shown to induce tumor progression in glioblastoma (Jiqiang Li, et al. International Journal of clinical experimental pathology. 2018), but its role in the TAMs within the TME is unclear.
**LY6E**	Lymphocyte Antigen 6 Family Member E has been shown to be highly expressed in several cancers in tumor cells and immune cells and associated with poor prognosis. Also, it can inhibit monocyte macrophage responsiveness and is involved in TGF-β signaling (Geeta Upadhyay. Frontiers in Immunology. 2019).
**IER5**	Immediate early response 5 has been shown to predict poor prognosis in glioma patients. Its correlation with immune suppressive microenvironment is not well established (Ziyun Wu, et al. Frontiers in Cell and Developmental biology. 2021).

**IFI27**	Interferon Alpha Inducible Protein 27 is an interferon stimulated gene and associated with INF-λ signaling that is known to be expressed in activated pro-inflammatory macrophages (Scott A. Read, et al. Frontiers immunology. 2019). However, in some cancers like pancreatic cancer it has been shown to be associated with immune suppressive macrophages and bad prognosis (Shu Huang, et al. Bioengineered. 2021). In lung cancer, it enhances chemo sensitivity and tumor eradication (Ang Yuan, et al. Scientific Reports. 2015). In glioma it has been shown to be expressed in immune suppressed microglia (Xianteng Cui, Frontiers in oncology. 2021).
**RNASE1**	Ribonuclease A Family Member 1 if expressed TREM2 high immune suppressive macrophages along other genes represents a special gene signature that predicts non responsiveness to immunotherapy (Donghai Xiong, et al. Nature communications. 2020).
**HSPA6**<br>**HSPA1B**	Heat Shock Protein Family A (Hsp70) Member 6 is reported to be predictive of prognosis in esophageal cancer. It has been associated with apoptosis suppression, tumor promotion within the TME in several cancers like ovarian cancer and hepatocellular cancer (Lin Wang, et al. International Immunopharmacology. 2020). However, in glioma it has been cited as one of the genes expressed within the immune TME and shown to be related with STAT3 signaling and immune suppression along with HSPAB1 (HSP70 family member 1B) (Maurizio Polano, et al. Cells. 2021; Gaetan Jego, et al. Cancers. 2020).
**DNAJB1**	DnaJ Heat Shock Protein Family (Hsp40) Member B1 inhibit MIG6 tumor suppressor gene and induce EGFR expression in Lung cancer (Soo-Yeon Park, et al. BBA-Molecular cell research. 2015). In glioma?
**HSPB1**	Heat Shock Protein Family B Member 1associated with autophagy and apoptosis and is upregulated in activated M1 and M2 macrophages. Its role dependent on the context (Paolo Fagone, et al. Inflammation Research. 2012).
**TIMP1**	TIMP Metallopeptidase inhibitor 1 expressed by TAMs in gliomas along with other genes associated with immune suppression but actual role within the TME is unknown (Wang Xiang MM, et al. Medicine. 2020).
**CXCL2**	CXC motif Chemokine Ligand 2. This chemokine is associated with angiogenesis, drug resistance and tumor growth in several cancers including brain cancers. It shares the same receptor as IL-8. CXCL2/CXCR2 is associated with ERK1/2 pathway activation (Fenghua Zhang, et al. Medicine. 2021; Ruth M. Urbantat, et al. Int J Mol Sci. 2021; Quan Zhang et al. Frontiers in Immunology. 2021).<br>Note that CXCL1, CXCL2, CXCL3 are known as powerful neutrophil attractant (Laila A. Al-Alwan, et al. The Journal of Immunology. 2013).
**RGCC**	Regulator of cell cycle.
**IL1B**	Interleukin 1 betta has an ambiguous role in some cancers immune suppressive and may in others have inflammatory responses (Houminji Chen, et al. 2021; Cedric Rebe and Francois Ghiringhelli. 2020). However, in gliomas it is related to M1 macrophages (Cong He, et al. Fontiers in cell and developmental biology. 2021).

**FCGBP**	Fc Fragment of IgG Binding Protein associated with M2 macrophage in ovarian cancer (Kai Wang, et al. Aging. 2021). It is a regulator of TGF-1- induced epithelial-mesenchymal transition that is associated with tumor progression and metastasis (Liguo Ye, et al. Journal of Translational Medicine. 2021), and is upregulated in glioma (Weihai Ning, et al. Journal of Molecular Neuroscience. 2020).
**VCAN**	Versican protein (proteoglycan) component of the extracellular matrix interacts and is produced by innate immune cells promoting myeloid cell anti-inflammatory responses and immune suppression in cancers (Thomas N. Wight et al. 2014; Mary Y. Chang et al. 2017; Thomas N. Wight, et al 2020).
**ISG15**	Interferon-stimulated gene 15 is INF-γ inducing chemokine and is essential for NK cells proliferations. In TMEs is dependent on the cancer some have shown its association with tumor suppression and others with M2 macrophages and tumor progression (Mei-Juan Zhou, et al. International Journal of Molecular Medicine. 2016; Ren-Hui Chen, et al. Frontiers in Immunology. 2020). In glioma, it is a tumor suppressor (Z. Du, et al. Biochemical and biophysical research communications. 2017).
**AREG**	Amphiregulin is associated with immune suppression and tumor progression (Moshit Lindzen, et al. 2021).
**CXCL3**	Has been shown to be related with M2 macrophages and associated with angiogenesis (Jennifer L. Owen and Mansour Mohamadzadeh. Frontiers in Physiology. 2013).
**CXCL8**	Or IL-8 known to be anti-inflammatory and associated with immunosuppressive TAMs (Zhiming Chen, et al. 2019; Tanwir Hasan, et al. 2019).
**IBSP**	Integrin Binding Sialoprotein has a role in wound healing and fibrosis but role in cancer immunity is not studied.
**CXCL10**	Chemokine associated with immune suppression (Kenji Shono, et al. Scientific reports. 2020; Imran G. House, et al. CCR. 2020; Guldner I.H. et al. Cell. 2020)
**HSP90AA1**	Heat Shock Protein 90 Alpha Family Class A Member 1 is associated with treatment resistance and its inhibition promote effective anti-tumor response (Kwon-Ho Song, et al. Nature Communications. 2020).
**CCL20**	Recently has been shown to be correlated with poor prognosis in glioma. It attracts immune suppressive macrophages to the TME and is induced by FDPS activation (Zhuo Chen, et al. Journal Cell and Molecular Medicine. 2020).
**CCL2**	It's function as a pro- or anti-inflammatory inducer in macrophages is context dependent (Martha Gschwandtner, et al. Frontiers in immunology. 2019).

Red Genes = immune suppressive; Green Genes = pro-inflammatory; Orange Genes = ambiguous; **Black genes** = nonspecific; Yellow Highlighted = markers of immune cell lineages and subsets. TME: Tumor microenvironment; TAMs: Tumor associated macrophages/microglia
